# Skin Wound Dressings Based on Biocompatible Nanofibers Loaded with the Mastoparan-Mo Peptide to Promote Regeneration and Control Infection in Skin Wounds

**DOI:** 10.1021/acsomega.6c02591

**Published:** 2026-10-05

**Authors:** Rubén D. Díaz-Martin, Angela Y. Becerra-Lovera, Guilherme Varejão S. Pasqual, Nathália Ribeiro Lobo, Stefhani Barcelos, Maria Victória Souto-Silva, Juliana Lott de Carvalho, Octávio Luiz Franco, Marcos L. Dias, Simoni Campos Dias

**Affiliations:** † Programa de Pós-graduação em Ciências Genômicas e Biotecnologia, 28106 Universidade Católica de Brasília, Brasília, Distrito Federal 71966-700, Brazil; ‡ Instituto de Macromoléculas Professora Eloisa Mano, 28125 Universidade Federal do Rio de Janeiro, Brasília, Rio de Janeiro 21941-598, Brazil; § Faculdade de Medicina, Universidade de Brasília, Brasília, Distrito Federal 70910-900, Brazil; ∥ S-Inova Biotech, Programa de Pós-Graduação em Biotecnologia, Universidade Católica Dom Bosco, Campo Grande, Mato Grosso do Sul 79117-010, Brazil

## Abstract

Skin wounds represent a major clinical challenge, particularly in chronic nonhealing cases associated with conditions such as diabetes, vascular disorders, prolonged immobilization, or trauma. These wounds involve a prolonged inflammatory process that impairs tissue regeneration, increases the risk of opportunistic infections, and further hinders healing, reducing quality of life and even threatening patient survival. Moreover, wound treatment significantly increases healthcare costs, making wound management a growing public health concern. In this study, we fabricated a series of skin wound dressings based on biocompatible nanofiber mats loaded with the antimicrobial and pro-regenerative peptide Mastoparan-Mo. We evaluated their physicochemical properties, antibacterial activity against clinically relevant bacteria, and pro-regenerative effects. These wound dressings exhibited well-defined ultrastructural organization, appropriate physicochemical properties, high hydrophilicity, and controlled-release behavior, supporting their potential as functional biomaterials. Moreover, they displayed antibacterial activity against several bacterial species commonly associated with skin infections and showed high biocompatibility in *in vitro* assays, promoting cell proliferation and motility in a model of primary human dermal fibroblasts. To assess the translational potential of these biomaterials, we used a model based on *ex vivo* human skin cultures derived from healthy skin explants, which preserve native tissue architecture and cellular heterogeneity. Using this model, we demonstrated that nanofiber wound dressings loaded with the Mastoparan-Mo peptide promote tissue regeneration in skin injuries within a physiologically relevant platform that closely mimics human skin in vivo.

## Graphical abstract

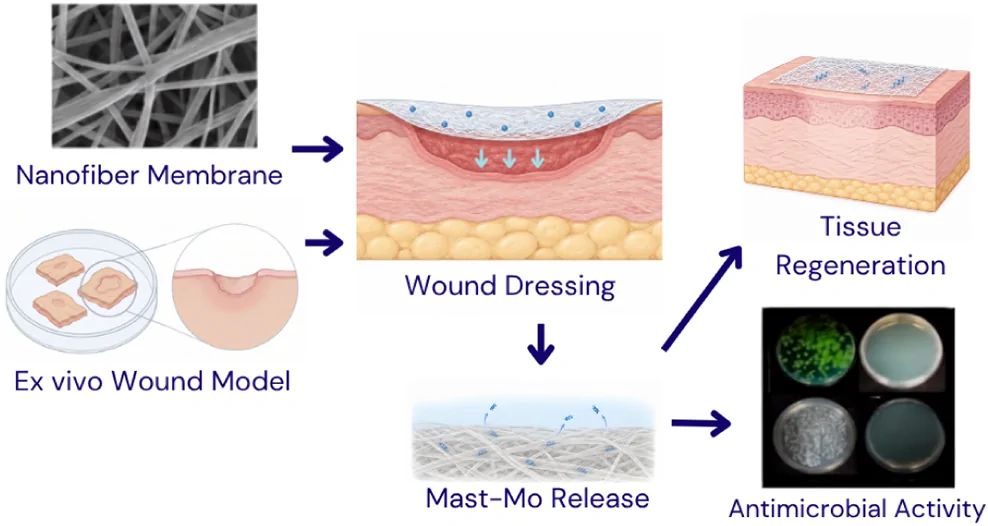

## Introduction

The skin serves as a protective barrier against mechanical, chemical, and microbiological insults, and its processes of regeneration and repair are highly coordinated. These processes involve precise regulation of inflammation, stimulation of cell proliferation, cell motility, and remodeling of the extracellular matrix to restore tissue integrity.^1^ Skin wounds pose a significant challenge due to complications associated with chronic wounds, such as nonhealing ulcers, or traumatic wounds, such as extensive burns, which are difficult to heal through conventional mechanisms. These complications increase the risk of clinical issues that can compromise individual health.^2,3^ Additionally, underlying metabolic and vascular disorders, as well as opportunistic infections, further exacerbate wound healing difficulties. This leads to prolonged treatment durations and rising healthcare costs, making the care and management of skin wounds an increasingly critical public health concern.^3,4^

In this context, the development of bioactive scaffolds for managing acute and chronic skin injuries is crucial for enhancing patient outcomes and quality of life. Ideally, these wound dressings should create conditions that promote cell proliferation, regulate the inflammatory response, exhibit low toxicity, and enable rapid biodegradability and absorption.^5,6^ Additionally, biomaterials functionalized with growth factors and other bioactive molecules have demonstrated promising results in accelerating epithelialization, reducing infections, and supporting efficient healing with minimal fibrotic scar formation.^3,6^

Several polymers have been identified as excellent candidates for developing biocompatible materials with high cellular affinity.^6,7^ These materials have shown significant potential in applications such as cartilage repair, treatment of ophthalmic lesions, and regeneration of injured skin.^8–12^ Their versatility stems from their tunable physicochemical properties and their ability to promote cell adhesion, proliferation, and differentiation.^6,7^ Furthermore, their biodegradability, suitability for the fabrication of controlled drug delivery systems, and favorable mechanical properties make them highly suitable for various bioengineering applications.^7^

One of the most promising polymers for the development of pro-regenerative scaffolds is poly­(vinyl alcohol) (PVA), a water-soluble, biocompatible polymer characterized by high degradability, cytocompatibility, and processability, along with excellent strength and elongation properties.^12,13^ This synthetic polymer has been approved by the Food and Drug Administration (FDA) for clinical use. Although PVA exhibits low intrinsic bioactivity, studies have shown that cells cultured in PVA hydrogels exhibit good adhesion and proliferation, highlighting its potential for tissue regeneration.^13,14^

Chitosan (CS) is a biopolymer with antibacterial, antioxidant, and immunomodulatory properties. It can interact with cells through surface receptor ligands, thereby mediating signaling pathways involved in cell adhesion and proliferation.^15^ Additionally, CS can activate and modulate the function of immune cells, such as neutrophils and macrophages, and facilitate the formation and organization of granulation tissue, a critical component of the wound-healing process.^15,16^ These characteristics, combined with its biocompatibility, biodegradability, nontoxicity, mucoadhesion, and broad-spectrum antibacterial activity, make chitosan a highly promising polymer for the development of healing dressings for treating skin wounds.^15^

The production of nanoscale wound dressings with pro-regenerative properties through electrospinning has attracted considerable attention. This technique enables the fabrication of polymeric nanofibers with a high surface-to-volume ratio, resulting in distinctive physicochemical properties. These fibers assemble into nonwoven networks characterized by interconnected porosity and high specific surface area, presenting an architecture that closely resembles the fibrillar organization of native collagen in the extracellular matrix (ECM). This structure provides cues that support cell adhesion, cytoskeletal organization, and proliferation, while facilitating nutrient and gas diffusion.^12,17^

In addition to their biomimetic structure, these systems typically exhibit high fiber density and reduced pore size, enhancing exudate absorption while serving as a physical barrier against bacterial infiltration. In addition, antimicrobial functionality can be introduced through different strategies that rely on the intrinsic properties of the polymers, including surface charge, pH responsiveness, and molecular architecture, as well as their ability to incorporate metals such as silver or metal oxides like zinc and copper, which can promote the generation of reactive oxygen species (ROS) under light irradiation or other physical stimuli.^18–20^

Furthermore, when these nanofiber platforms incorporate bioactive agents, such as antibiotics or antimicrobial peptides (AMPs), they function as delivery systems for the controlled release of bioactive molecules, improving local bioavailability, protecting active compounds from premature degradation, and maintaining effective concentrations at the target site.^21,22^

Within this framework, nanofiber membranes based on poly­(vinyl alcohol) and chitosan (PVA/CS) represent a particularly promising platform. Their combination of hydrophilicity, mechanical strength, biodegradability, porosity, and wettability is key to supporting tissue regeneration processes.^12^ Consistent evidence from various experimental models indicates that PVA/CS-based scaffolds, either alone or combined with other biopolymers, enhance cell proliferation and accelerate skin regeneration in vivo, even under diverse pathological conditions, including metabolic disorders and extensive burn injuries, suggesting the strong potential of PVA/CS nanofiber systems as pro-regenerative platforms for tissue engineering applications.^12,20^

Several AMPs have been observed to promote skin tissue repair through various mechanisms, including the modulation of cytokine production, induction of cell migration, stimulation of cell proliferation, and regulation of angiogenesis.^23^ These properties offer a new and promising strategy for treating nonhealing or traumatic wounds.^23,24^

In this context, mastoparans, a group of short and amphipathic peptides derived from wasp venom, exhibit antibacterial activity through a primarily membranolytic mechanism and show activity against biofilms and clinically relevant pathogens, showing therapeutic potential, which is particularly relevant in chronic wounds.^25–27^

Among these, Mastoparan-MO (Mast-MO), a synthetic analog of mastoparan L, has been shown to enhance leukocyte migration while suppressing the expression of pro-inflammatory factors such as TNF-α and IL-6, thereby mitigating the damage caused by excessive inflammatory responses and promoting tissue regeneration.^28^ Recently, we demonstrated that Mast-MO can stimulate the proliferation and migration of human fibroblasts *in vitro*. This process was accompanied by gene expression signatures associated with skin regeneration, highlighting the pro-regenerative properties of this peptide.^29^

The use of alternative models has enabled the development of more ethical and physiologically relevant approaches for evaluating biomaterials, reducing dependence on animal experimentation while allowing controlled, reproducible, and cost-effective assessment of biological responses under well-defined experimental conditions.^30^

In this context, models such as the human organotypic model of human skin (hOSEC), allow the study of tissue responses in a system that preserves the anatomical and physiological organization of human skin, including epidermal and dermal layers, cell–cell interactions, and extracellular matrix components. This provides a more clinically relevant platform for evaluating the impact of biomaterials on regenerative processes, with improved translational relevance, reduced variability, and better control over experimental conditions compared to conventional *in vivo* models.^31–33^

In this study, we fabricated a series of skin wound dressings based on biocompatible nanofiber mats loaded with the antimicrobial and pro-regenerative peptide Mast-Mo. We evaluated their physicochemical properties, antibacterial activity against clinically relevant bacteria, and *in vitro* pro-regenerative effects. Furthermore, employing an alternative model based on *ex vivo* human skin cultures derived from healthy explants, which retain native tissue architecture, we assessed the pro-regenerative capacity of these nanofiber wound dressings in a translationally relevant human skin model.

Physicochemical characterization revealed that the nanofiber membranes exhibit properties suitable for the development of wound healing biomaterials. Likewise, these mats demonstrated antibacterial activity, high biocompatibility, and the ability to induce cell proliferation and motility in primary human skin fibroblasts. Furthermore, wound-healing assays using an hOSEC-model, showed that this biomaterial can promote human skin regeneration under *ex vivo* conditions. These results suggest that this strategy can integrate infection control with enhanced skin repair and regeneration within a single biomaterial platform.

## Results

### Morphological Characteristics

The ultrastructural analysis reveals that both materials (PVA/CS-73 and PVA/CS-91, with or without the peptide) form a fibrous mat composed of randomly oriented, bead-free, smooth, and uniformly shaped cylindrical nanofibers. The nanofibers were tightly packed and highly entangled, without the formation of nodes (Figure 1).

**Figure 1:**
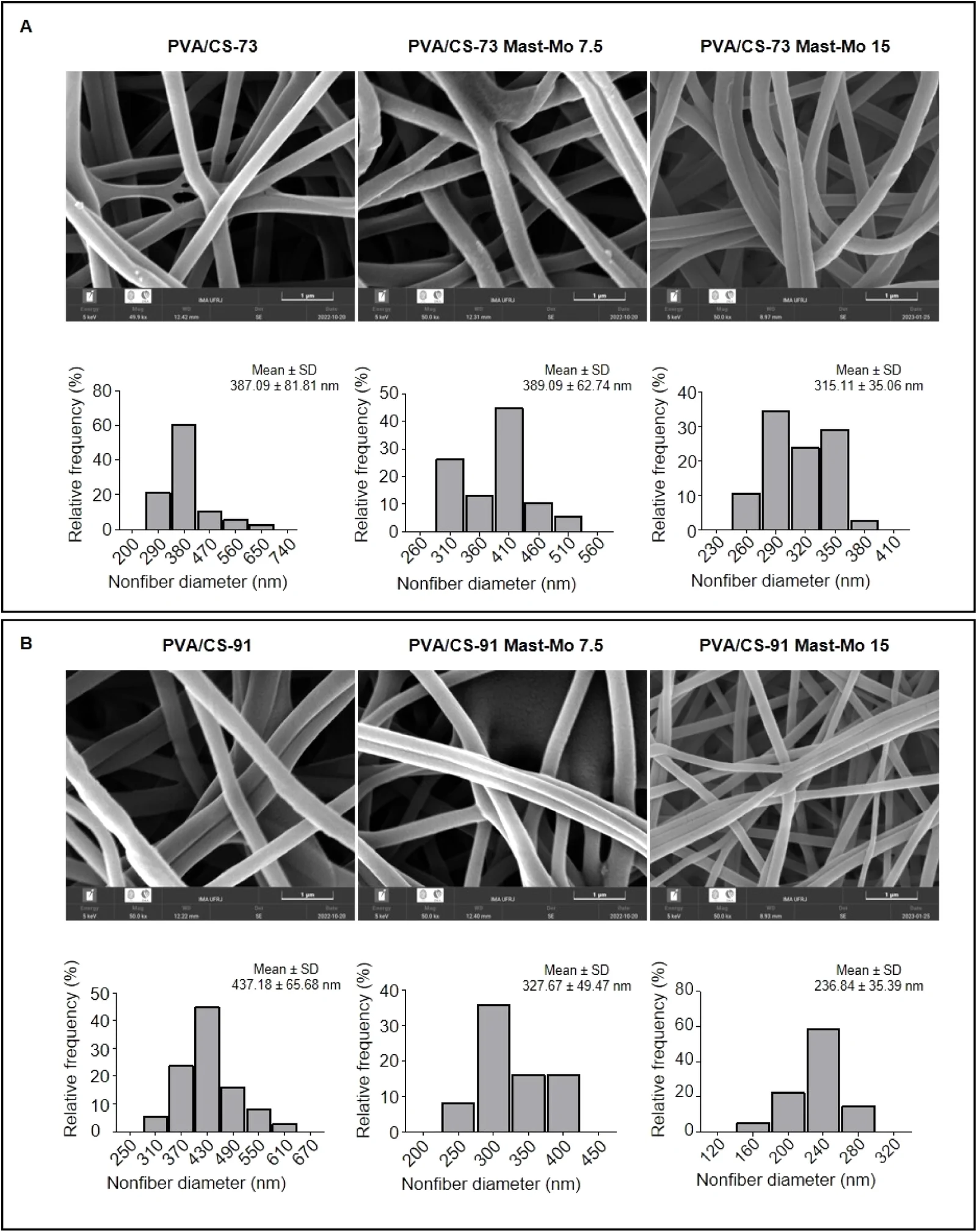
Morphology and diameter distribution of electrospun nanofibers. SEM images and histograms of the diameter distribution of (A) PVA/CS-73 nanofibers loaded with the Mast-Mo peptide at 0, 7.5, and 15 μM (upper panel), and (B) PVA/CS-91 nanofibers loaded with the Mast-Mo peptide at 0, 7.5, and 15 μM (lower panel). Scale bar = 1 μm. In both A and B, representative SEM images are shown. Measurements were obtained from a total of 50 nanofibers across 10 fields at 10,000× magnification. Morphometric analysis was performed using ImageJ software.

In PVA/CS-73, the nanofibers without the Mast-Mo peptide and those containing 7.5 and 15 μM of the Mast-Mo peptide (PVA/CS-73, PVA/CS-73 Mast-Mo 7.5, and PVA/CS-73 Mast-Mo 15, respectively) exhibited average diameters of 387.09 ± 81.81, 389.09 ± 62.74, and 315.11 ± 35.06 nm, respectively (Figure 1A). Statistical analysis of PVA/CS-73 nanofiber diameter following Mast-Mo incorporation indicates a shift in mean diameter at 15 μM; however, this effect was not statistically significant (Figure S1A; p = 0.065), suggesting only a trend rather than a definitive change.

### Chemical Characterization

The chemical composition analysis showed that both types of nanofibers (PVA/CS-73 and PVA/CS-91, Figure 2A, left and right panels, respectively) exhibited characteristic bands corresponding to the functional groups present in PVA and CS.

**Figure 2:**
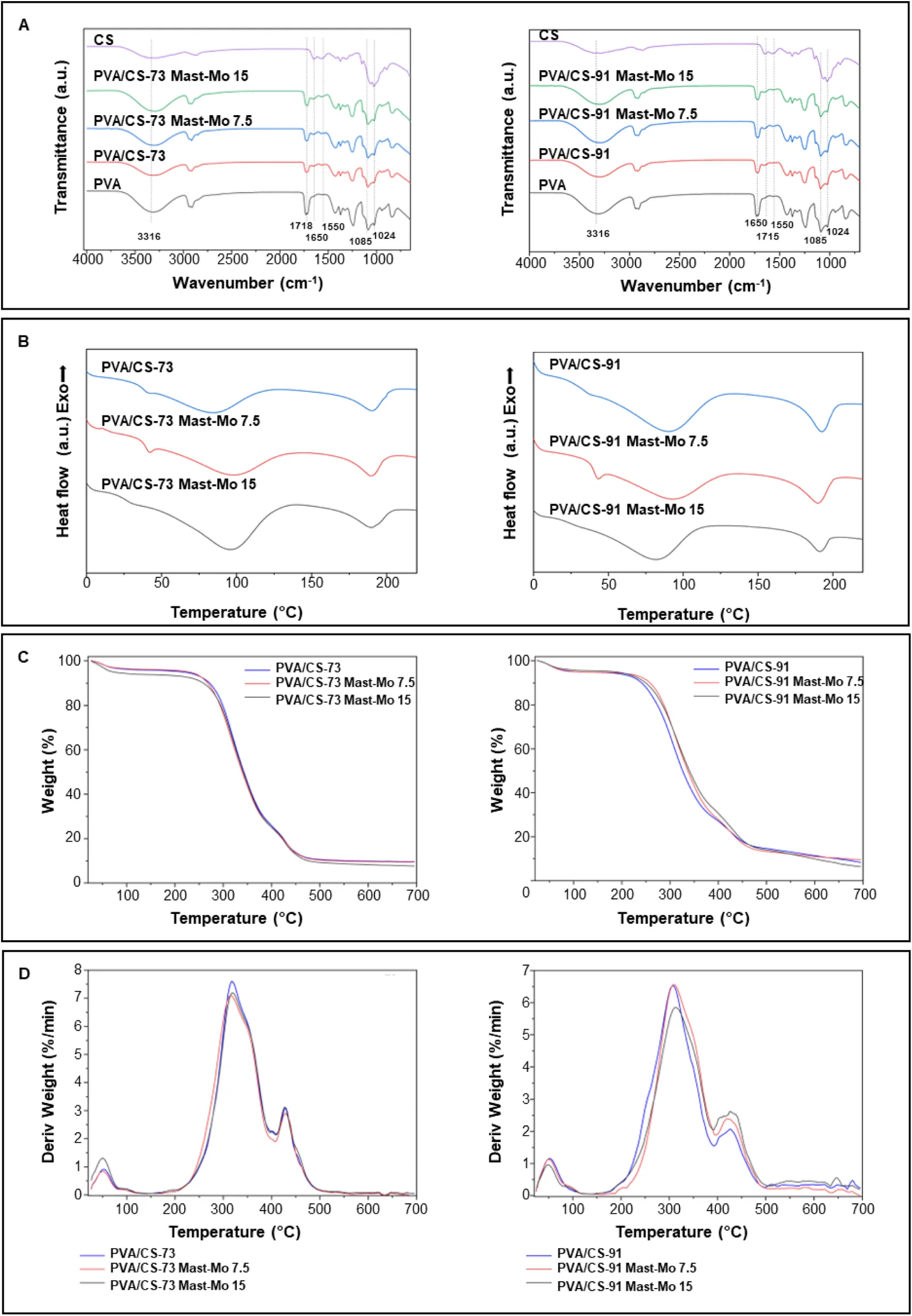
Physicochemical characterization of the electrospun nanofibers. (A) FTIR spectra of PVA/CS-73 (left panel) and PVA/CS-91 (right panel) nanofibers containing different amounts of the Mast-Mo peptide. (B) DSC thermograms of PVA/CS-73 (left panel) and PVA/CS-91 (right panel) nanofibers containing different amounts of the Mast-Mo peptide. (C) TG thermographs of PVA/CS-73 (left panel) and PVA/CS-91 (right panel) nanofibers containing different amounts of the Mast-Mo peptide. (D) DTG thermographs of PVA/CS-73 (left panel) and PVA/CS-91 (right panel) nanofibers containing different amounts of the Mast-Mo peptide.

Bands associated with C–O stretching were identified at 1085 and 1024 cm^–1^, while a peak near 840 cm^–1^, assigned to out-of-plane C–H bending, confirmed that the main polymer structure was retained after electrospinning.

Aliphatic C–H stretching bands in the 2940–2900 cm^–1^ range, corresponding to CH_2_ (∼2941 cm^–1^) and CH_2_ (∼2915 cm^–1^), as well as the carbonyl band (C=O) from residual acetate groups of PVA (1715–1733 cm^–1^), were also identified in the PVA/CS samples. Likewise, amide I (∼1650 cm^–1^) and amide II (1580–1550 cm^–1^) bands, attributed to CS, were present in these materials.

All nanofiber mats exhibited a broad and intense band at approximately 3316 cm^–1^, attributed to the combined stretching of hydroxyl (O–H) and amine (N–H) groups. This band shifted to approximately 3300 cm^–1^ compared to the pure components, suggesting the formation of hydrogen bonds between PVA and chitosan chains. Similarly, a shift of the PVA C–O stretching peak from 1090 to 1085 cm^–1^ further supported the presence of these interactions. Notably, the higher transmittance observed in the PVA/CS samples for the asymmetric CH_2_ and CH_2_ stretching modes (∼2941 cm^–1^ and ∼2915 cm^–1^, respectively) suggests molecular reorganization and enhanced intermolecular interactions.

The incorporation of the Mast-Mo peptide (7.5 and 15 μM) induced concentration-dependent spectral changes, indicating specific interactions of the peptide with the polymer matrix. A shift in the ν­(O–H)/ν­(N–H) region to 3290–3295 cm^–1^, accompanied by a progressive decrease in transmittance, suggests the formation of new hydrogen bonds between peptide functional groups (−COOH, −NH_2_) and the polymeric components.

Additionally, a concentration-dependent decrease in the intensity of the ν­(C–O) vibrations (1085–1024 cm^–1^), with a maximum effect at 15 μM, suggests further reorganization of hydrogen bonding within the matrix.

Regarding the amide bands, a progressive increase in the intensity of amide I (≈1650 cm^–1^) was observed, indicating a higher contribution of amino groups, while a reduction in transmittance in the amide II region (1560–1580 cm^–1^) was detected.

Thermograms of the PVA/CS-73 and PVA/CS-91 wound dressings are shown in Figure 2B. In the PVA/CS-73 materials (left panel), the enthalpy of fusion (ΔHf_1_) increased progressively with increasing peptide concentration (Table S1), indicating greater retention of polar volatiles. By contrast, the PVA/CS-91 materials (Figure 2B, right panel) exhibited a nonmonotonic response. In the PVA/CS-91 with Mast-Mo at 7.5 μM sample, ΔHf_1_ decreased to 51.4 mJ/mg compared to 98.3 mJ/mg in the control, whereas in PVA/CS-91 with Mast-Mo at 15 μM, ΔHf_1_ increased to 134 mJ/mg. Additionally, the peak temperature decreased to 81.9 °C in the peptide-functionalized samples.

The peak at 190 °C, corresponding to the melting of PVA crystalline domains, remained stable across all PVA/CS samples, indicating that the addition of chitosan did not significantly affect the thermal stability of the PVA crystallites. However, the incorporation of Mast-Mo modified the crystallinity parameters (ΔHf_2_) in both formulations. In PVA/CS-73, ΔHf_2_ slightly decreased at 7.5 μM (21.3 vs 22.7 mJ/mg in the control) and increased at 15 μM (24.7 mJ/mg), whereas in PVA/CS-91, ΔHf_2_ decreased markedly at 7.5 μM and increased to 32.9 mJ/mg at 15 μM.

Thermogravimetric analysis of the PVA/CS-73 and PVA/CS-91 wound dressings is shown in Figure 2C and D and in the Table S2. This analysis revealed a consistent three-stage degradation profile. The first stage, centered near 50 °C, corresponded to moisture evaporation and accounted for ≤7% mass loss. The second and dominant stage, reflected by *T*_max1_ (308–329 °C), accounted for 80–87% mass loss. The PVA/CS-73 series displayed higher T_onset_ (271–283 °C) and *T*_max1_ (325–329 °C) values compared to the PVA/CS-91 series (T_onset_ = 248–262 °C; *T*_max1_ = 308–318 °C), indicating improved thermal stability. The third stage, marked by *T*_max2_ (427–432 °C), exhibited a narrow temperature range and high reproducibility across all samples, confirming that this thermal event was not affected by variations in the PVA:CS ratio or peptide content.

Char residue at 700 °C ranged from 7 to 10%, with the PVA/CS-73 control yielding 10% and the 9:1 control yielding 8%, consistent with the known char-forming behavior of chitosan in contrast to the near-complete volatilization of PVA. Samples containing 26 μg peptide exhibited 7% char residue; however, this minor reduction did not represent a systematic trend. The observed differences among formulations were small and fell within the instrumental and methodological error margins of TGA.

### Swelling Behavior, Surface Wettability, and *In Vitro* Degradability

The swelling test revealed that all PVA/CS-73 wound dressings, regardless of the presence of the Mast-Mo peptide, exhibited a weight gain of approximately twice their dry weight (PVA/CS-73 Mast-Mo at 0, 7.5, and 15 μM: 2.12 ± 0.04, 2.20 ± 0.25, and 2.15 ± 0.05 times their dry weight, respectively; Figure 3A, left panel).

**Figure 3:**
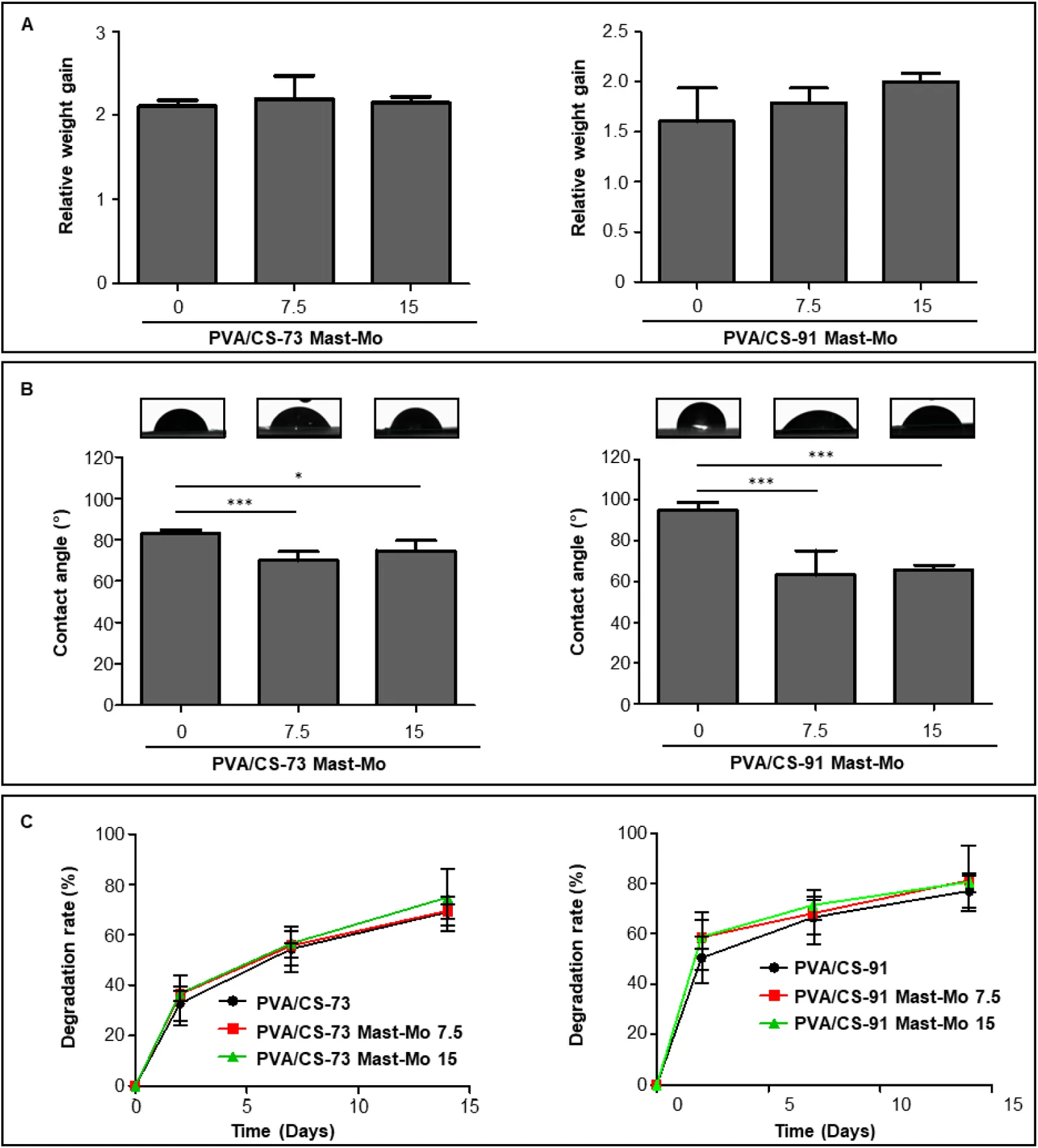
Swelling behavior, wettability, and degradation profile. (A) Weight gain ratio of nanofiber mats relative to their dry weight, measured at 24 h for PVA/CS-73 (left panel) and PVA/CS-91 (right panel) containing different amounts of the Mast-Mo peptide. (B) Water contact angle measurements of PVA/CS-73 nanofiber wound dressings (left panel) and PVA/CS-91 wound dressings (right panel) containing different amounts of the Mast-Mo peptide. (C) Nanofiber mat degradation (%) for PVA/CS-73 (left panel) and PVA/CS-91 (right panel) containing different amounts of the Mast-Mo peptide, measured at 2, 7, and 14 days. Data are presented as mean ± SD from three independent experiments. Group comparisons were performed using ANOVA followed by Tukey’s post hoc test versus the control (Ctrl); asterisks indicate significance (*p ≤ 0.05; ***p ≤ 0.001).

By contrast, all PVA/CS-91 wound dressings, with or without the Mast-Mo peptide, showed a weight gain of approximately 1.8 times their dry weight (PVA/CS-91 Mast-Mo at 0, 7.5, and 15 μM: 1.70 ± 0.20, 1.81 ± 0.10, and 1.89 ± 0.26 times their dry weight, respectively; Figure 3A, right panel).

Wettability analysis by water contact angle measurements showed that PVA/CS-73 wound dressings in the absence of the Mast-Mo peptide exhibited contact angles of 82.89 ± 1.82° (Figure 3B, left panel), whereas the addition of the Mast-Mo peptide at 7.5 and 15 μM resulted in a significant reduction in the contact angle (69.91 ± 4.30°, *p* ≤ 0.001, and 74.70 ± 4.97°, *p* ≤ 0.05, respectively).

Similarly, it was observed that PVA/CS-91 wound dressings, in the absence of the peptide, presented a contact angle of 94.88 ± 3.94° (Figure 3B, right panel), whereas peptide incorporation significantly reduced this value (63.25 ± 11.91° at 7.5 μM and 65.8 ± 2.28° at 15 μM; both *p* ≤ 0.001), suggesting that peptide incorporation enhances the hydrophilicity of the nanofiber wound dressings.

The biodegradability analysis revealed that PVA/CS-73 nanofiber with Mast-Mo at 0, 7.5, and 15 μM (Figure 3C, left panel), lost an average of approximately 34% of their initial mass by day 2 (32.78 ± 5.81%, 36.40 ± 6.53%, and 36.81 ± 6.62%, respectively). After 7 days, mass loss reached approximately 55% (54.41 ± 11.17%, 55.72 ± 1.67%, and 56.62 ± 1.64%, respectively). By day 14, degradation progressed to approximately 70% of the initial mass (69.14 ± 3.17%, 69.57 ± 3.26%, and 74.91 ± 3.01%, respectively).

In contrast, PVA/CS-91 nanofibers, with 0, 7.5, and 15 μM of the Mast-Mo peptide, exhibited more pronounced degradation (Figure 3C, right panel), with an average mass loss of approximately 55% by day 2 (50.37 ± 6.98%, 58.65 ± 1.84%, and 58.77 ± 3.46%, respectively). By day 7, these nanofibers exhibited a slower degradation rate, with mass loss reaching approximately 70% (66.57 ± 8.88%, 68.14 ± 5.21%, and 71.40 ± 5.38%, respectively). Notably, all PVA/CS-91 nanofiber membranes lost around 80% of their initial mass by day 14 (77.06 ± 5.25%, 81.27 ± 5.04%, and 80.66 ± 1.23%, respectively).

### Antibacterial Activity

The evaluation of antibacterial activity against *P. aeruginosa* (Figure 4A) showed that treatment with PVA/CS-73 and PVA/CS-91 wound dressings loaded with 7.5 μM of the peptide resulted in only a slight reduction in bacterial viability. By contrast, the material loaded with 15 μM of the Mast-Mo peptide induced a significant decrease of more than 95% in bacterial viability (*p* ≤ 0.001), whereas peptide-free nanofibers showed no significant effect.

**Figure 4:**
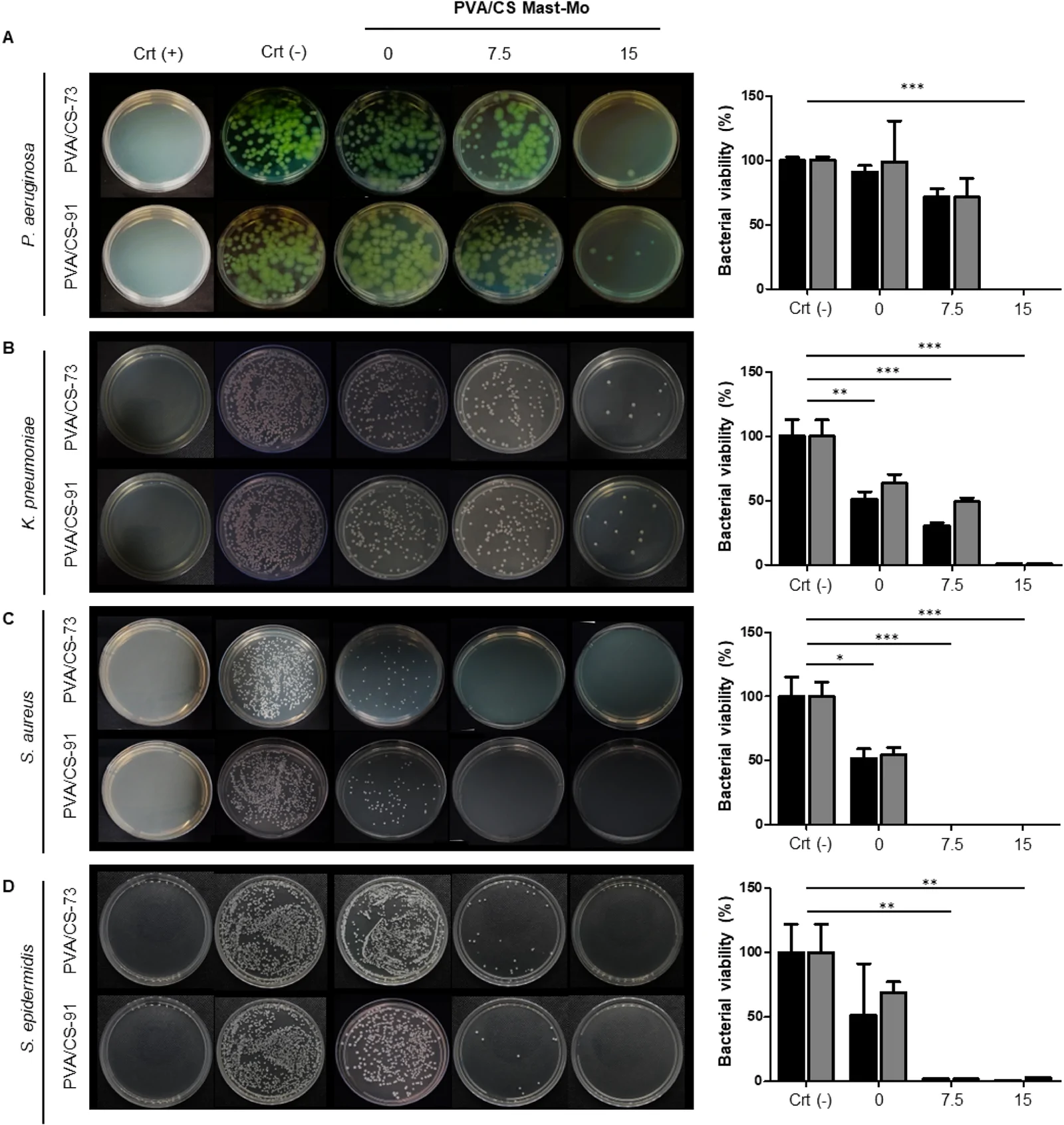
Antibacterial activity. Representative images of plates used for CFU counting are shown on the left, while bacterial viability (%) after treatment with nanofiber wound dressings containing different amounts of Mast-Mo peptide is presented on the right. (A) *P. aeruginosa*; (B) *K. pneumoniae*; (C) *S. aureus*; (D) *S. epidermidis*. Black bars represent PVA/CS-73 and gray bars represent PVA/CS-91. Data are presented as mean ± SD from three independent experiments. Group comparisons were performed using the Kruskal–Wallis test followed by Dunn’s test with Bonferroni correction versus control (Crt, −); asterisks indicate significance (*p ≤ 0.05; **p ≤ 0.01; ***p ≤ 0.001).

On the other hand, both PVA/CS-73 and PVA/CS-91 nanofiber wound dressings without Mast-Mo peptide loading produced a significant reduction in the bacterial viability of *K. pneumoniae*, exceeding 35% (*p* ≤ 0.01). Likewise, nanofiber wound dressings (PVA/CS-73 and PVA/CS-91) loaded with 7.5 or 15 μM of the Mast-Mo peptide further reduced bacterial viability against *K. pneumoniae* (Figure 4B). This reduction in bacterial viability exceeded 50% for both materials at 7.5 μM Mast-Mo (*p* ≤ 0.001), whereas wound dressings loaded with 15 μM Mast-Mo achieved reductions greater than 98% for both PVA/CS-73 and PVA/CS-91 (*p* ≤ 0.001).

By contrast, the antibacterial activity of PVA/CS-73 and PVA/CS-91 wound dressings against *S. aureus* revealed that both types of nanofibers loaded with 7.5 or 15 μM of the Mast-Mo peptide significantly reduced bacterial viability by more than 95% (*p* ≤ 0.001) (Figure 4C). Notably, both types of nanofibers, even in the absence of the Mast-Mo peptide, induced a significant reduction of more than 40% *in* *S. aureus* viability (*p* ≤ 0.05).

Similarly, the antibacterial activity of PVA/CS-73 and PVA/CS-91 nanofiber wound dressings against *S. epidermidis* showed that materials loaded with the Mast-Mo peptide at both 7.5 and 15 μM produced a significant reduction in bacterial viability of more than 90% (*p* ≤ 0.01) (Figure 4D). However, when analyzing the effect of the PVA/CS-73 and PVA/CS-91 nanofiber wound dressings in the absence of the Mast-Mo peptide, a reduction of more than 30% in bacterial viability was observed. Despite this marked trend, this reduction was lower than that observed for the peptide-loaded materials and was not significantly different compared to the negative control (TSB medium).

### Biocompatibility

To establish the safety range of the bioactive peptide, we first evaluated the cytotoxic potential of the antimicrobial peptide Mast-Mo on primary human dermal fibroblasts (hFibs) at concentrations ranging from 0 to 50 μM. No cytotoxic effects were observed at 5, 10, and 20 μM. In contrast, a significant and progressive decrease in cell viability was detected at 30, 40, and 50 μM, with reductions of 42.02 ± 11.4%, 61.3 ± 7.60%, and 67.7 ± 6.34%, respectively (*p* < 0.001). These findings indicate that concentrations below 20 μM are likely safe for use in the fabrication of wound dressings (Figure 5A).

**Figure 5:**
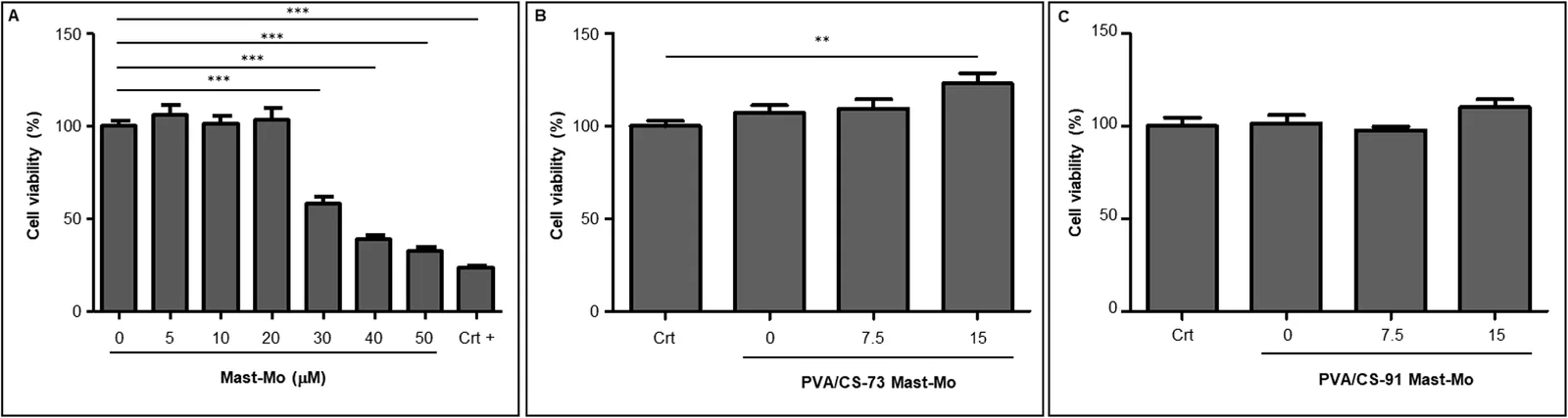
Biocompatibility. (A) The cytotoxic potential of the Mast-Mo peptide at 0, 5, 10, 20, 30, 40, and 50 μM was evaluated in hFibs cells. The biocompatibility of nanofiber wound dressings loaded with 0, 7.5, and 15 μM of the Mast-Mo peptide was assessed in hFib cells using 10 mg/mL of each material: (B) PVA/CS-73 and (C) PVA/CS-91. Data are presented as mean ± SD (*n* = 3). One-way ANOVA with Tukey’s post hoc test was applied; asterisks indicate differences versus the control (**p ≤ 0.01; ***p ≤ 0.001).

The biocompatibility evaluation in hFibs cells showed that neither PVA/CS-73 nor PVA/CS-91 wound dressings loaded with the peptide at concentrations of 7.5 or 15 μM caused significant changes in cell viability, even at nanofiber mat concentrations as high as 10 mg/mL (Figure 5B and C).

Notably, the PVA/CS-73 wound dressing with Mast-Mo at 15 μM, significantly increased cell viability compared to the unexposed control group (*p* ≤ 0.01), suggesting a potential pro-proliferative effect (Figure 5B). Furthermore, nanofibers without the bioactive peptide did not induce any significant changes in human fibroblast viability when compared with the nonexposed control group.

### In Vitro Cell Migration and Proliferation

Wound-closure analysis showed that the treatment with PVA/CS-73 and PVA/CS-91 wound dressings loaded with 7.5 or 15 μM of the Mast-Mo peptide significantly reduced the lesion area compared with the control group treated with vehicle medium (Figure 6A and B).

**Figure 6:**
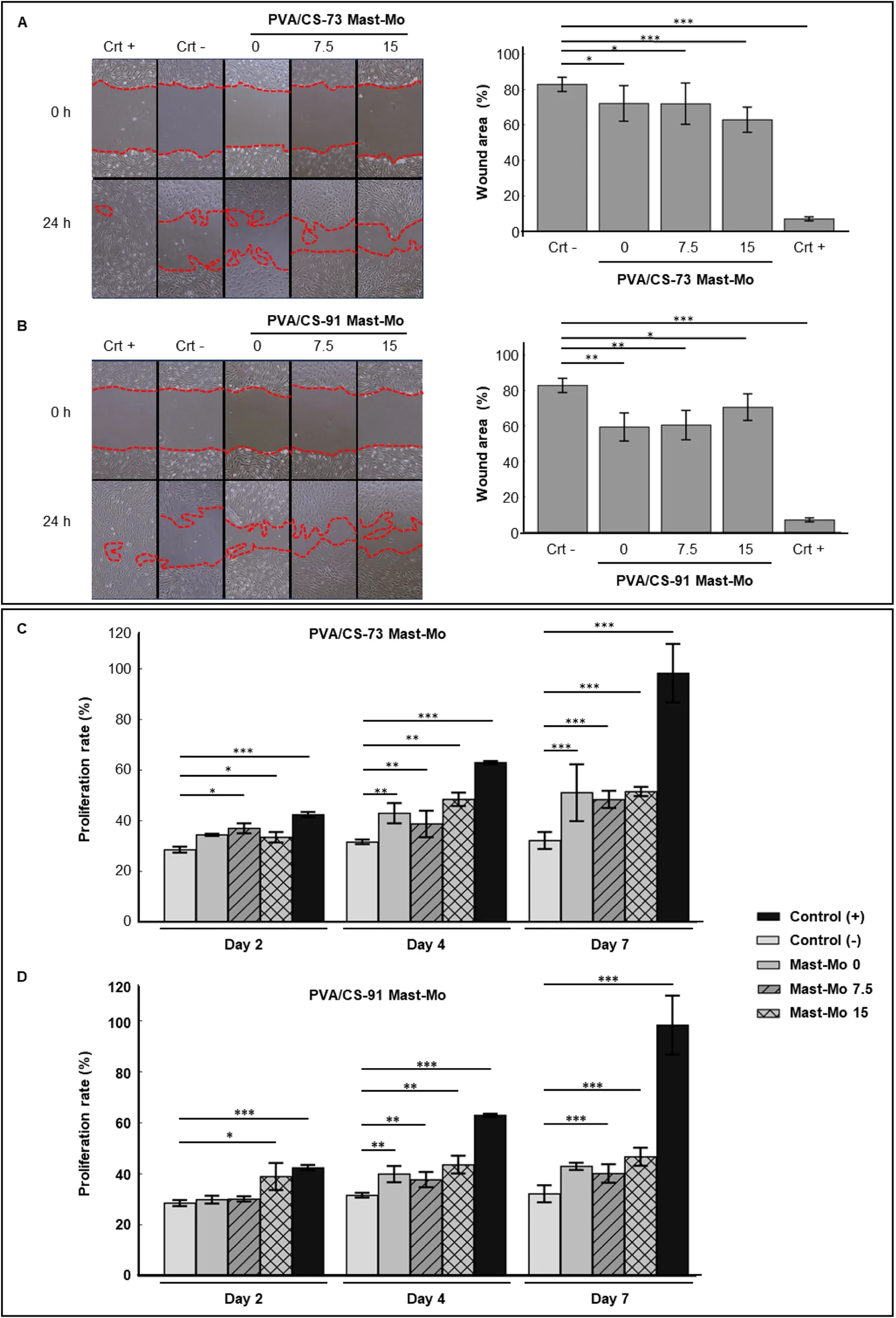
Cell migration and proliferation. Cell migration in hFib cells at 0 and 24 h was quantified by image analysis (left panel). (A) PVA/CS-73 and (B) PVA/CS-91. Data are mean ± SD from three independent experiments (right panel). The proliferative effect of Mast-Mo–loaded nanofiber dressings was evaluated in hFib at 2, 4, and 7 days: (C) PVA/CS-73 and (D) PVA/CS-91. Data are presented as mean ± SD from three independent experiments. Comparisons were performed using one-way ANOVA followed by Tukey’s post hoc test; significant differences versus the control are indicated by asterisks (*p ≤ 0.05; **p ≤ 0.01; ***p ≤ 0.001).

The wound area decreased from 82.82% ± 2.60 in the control group to 70.12% ± 15.62 (PVA/CS-73 with Mast-Mo at 7.5 μM, *p* ≤ 0.05), 62.89% ± 4.23 (PVA/CS-73 with Mast-Mo at 15 μM, *p* ≤ 0.001), 60.41% ± 5.11 (PVA/CS-91 with Mast-Mo at 7.5 μM, *p* ≤ 0.01), and 70.53% ± 7.98 (PVA/CS-91 with Mast-Mo at 15 μM, *p* ≤ 0.05).

Notably, PVA/CS-73 and PVA/CS-91 nanofiber wound dressings without the peptide also significantly reduced the lesion area, which may be attributed to the intrinsic properties of the PVA/CS matrix. In the absence of the peptide, the lesion area was reduced to 72.10% ± 10.27 (PVA/CS-73, *p* ≤ 0.05) and 44.00% ± 6.96 (PVA/CS-91, *p* ≤ 0.01).

In the proliferation assay, it was observed that after 2 days of treatment, both nanofiber wound dressings with Mast-Mo at 15 μM significantly increased cell proliferation compared with the control group treated with vehicle medium (*p* ≤ 0.05) (Figure 6C and D). The proliferation rate increased from 28.69% ± 1.17 in the control group to 33.46% ± 2.08 for PVA/CS-73 Mast-Mo at 15 μM and 38.89% ± 5.29 for PVA/CS-91 Mast-Mo at 15 μM.

At both 4 and 7 days of treatment, PVA/CS-73 and PVA/CS-91 nanofibers loaded with Mast-Mo at 7.5 and 15 μM also induced a significant increase in cell proliferation compared with the control group. For PVA/CS-73 nanofiber wound dressings (Figure 6C), cell proliferation increased at day 4 from 31.63% ± 0.94 in the control group to 38.71% ± 5.23 (Mast-Mo at 7.5 μM, *p* ≤ 0.01) and 48.45% ± 2.64 (Mast-Mo at 15 μM, *p* ≤ 0.001). At day 7, proliferation further increased to 48.36% ± 3.36 (Mast-Mo 7.5 μM, *p* ≤ 0.01) and 51.49% ± 1.80 (Mast-Mo at 15 μM, *p* ≤ 0.001).

Similarly, PVA/CS-91 nanofiber wound dressings (Figure 6D) showed enhanced cell proliferation at day 4, reaching 37.69% ± 2.96 and 43.58% ± 3.48 (Mast-Mo at 7.5 and 15 μM respectively, *p* ≤ 0.01). At day 7, proliferation reached 40.08% ± 3.64 and 46.71% ± 3.52 (Mast-Mo at 7.5 and 15 μM respectively, *p* ≤ 0.01).

Notably, peptide-free nanofiber (PVA/CS-73 and PVA/CS-91) also induced a significant increase in cell proliferation at 2, 4, and 7 days. At day 2, PVA/CS-73 nanofiber significantly increased cell proliferation to 34.38% ± 0.47% (*p* ≤ 0.05). Likewise, at days 4 and 7, proliferation reached 42.94% ± 4.03% and 39.90% ± 3.18% at day 4, and 51.03% ± 11.20% and 42.94% ± 1.42% at day 7 for PVA/CS-73 and PVA/CS-91, respectively (*p* ≤ 0.01).

### Ex-Vivo Wound Healing Assays

The evaluation of the pro-regenerative potential of PVA/CS nanofiber wound dressings with the Mast-Mo peptide in an *ex vivo* organotypic model of human skin indicated that treatment with PVA/CS-73 Mast-Mo at 15 μM resulted in a significant reduction in lesion area compared with the control group, which received only the vehicle (from 75.21% ± 10.78 in the control group to 51.11% ± 15.74 in the PVA/CS-73 with Mast-Mo at 15 μM group; *p* ≤ 0.05; Figure 7A).

**Figure 7:**
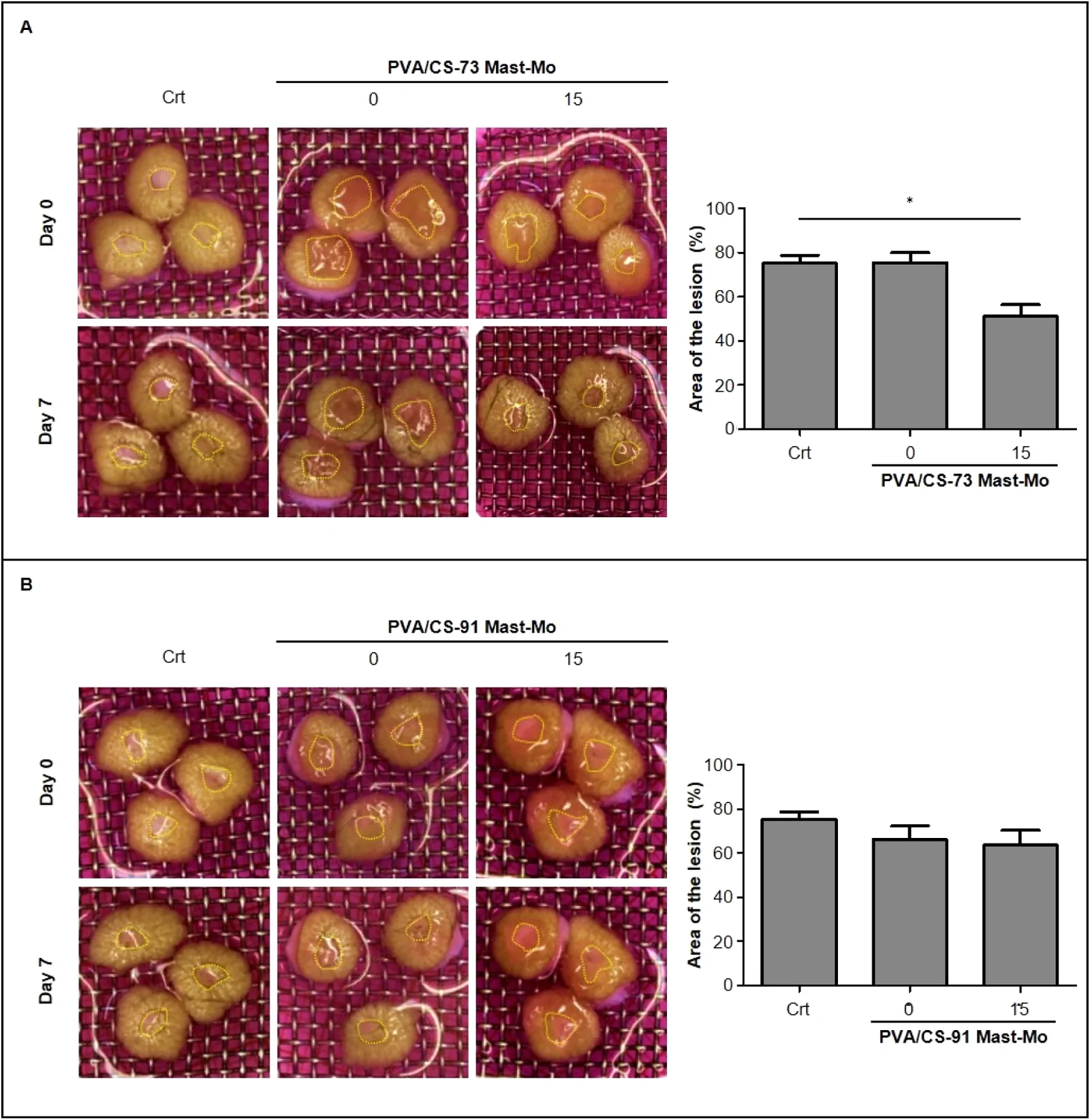
Wound healing in an *ex vivo* hOSEC model. Skin explants were treated with nanofiber wound dressings loaded with the Mast-Mo peptide (15 μM) for 7 days. Re-epithelialization was quantified by measuring lesion closure in the explants using ImageJ software (left panel): (A) PVA/CS-73 and (B) PVA/CS-91. Data are presented as mean ± SD from three independent experiments (right panel). Comparisons among groups were performed using one-way ANOVA followed by Tukey’s post hoc test. Asterisks indicate significant differences versus the control (*p ≤ 0.05).

By contrast, treatment with PVA/CS-91 Mast-Mo at 15 μM produced a slight trend toward lesion area reduction; however, this difference was not statistically significant (from 75.21% ± 10.78 in the control group to 63.56% ± 20.05 in the PVA/CS-91 with Mast-Mo at 15 μM group; Figure 7B).

### Peptide Release Profile and Kinetic Modeling

The analysis of the release profile of nanofiber wound dressings containing 15 μM of the Mast-Mo peptide showed that Mast-Mo release from PVA/CS-73 (Figures 8 and S2) reached 32.41 ± 8.75% within the first 24 h. Subsequently, peptide release exceeded 60% by 48 h (66.33 ± 5.43%) and reached 82.41 ± 18.42% at 120 h.

**Figure 8:**
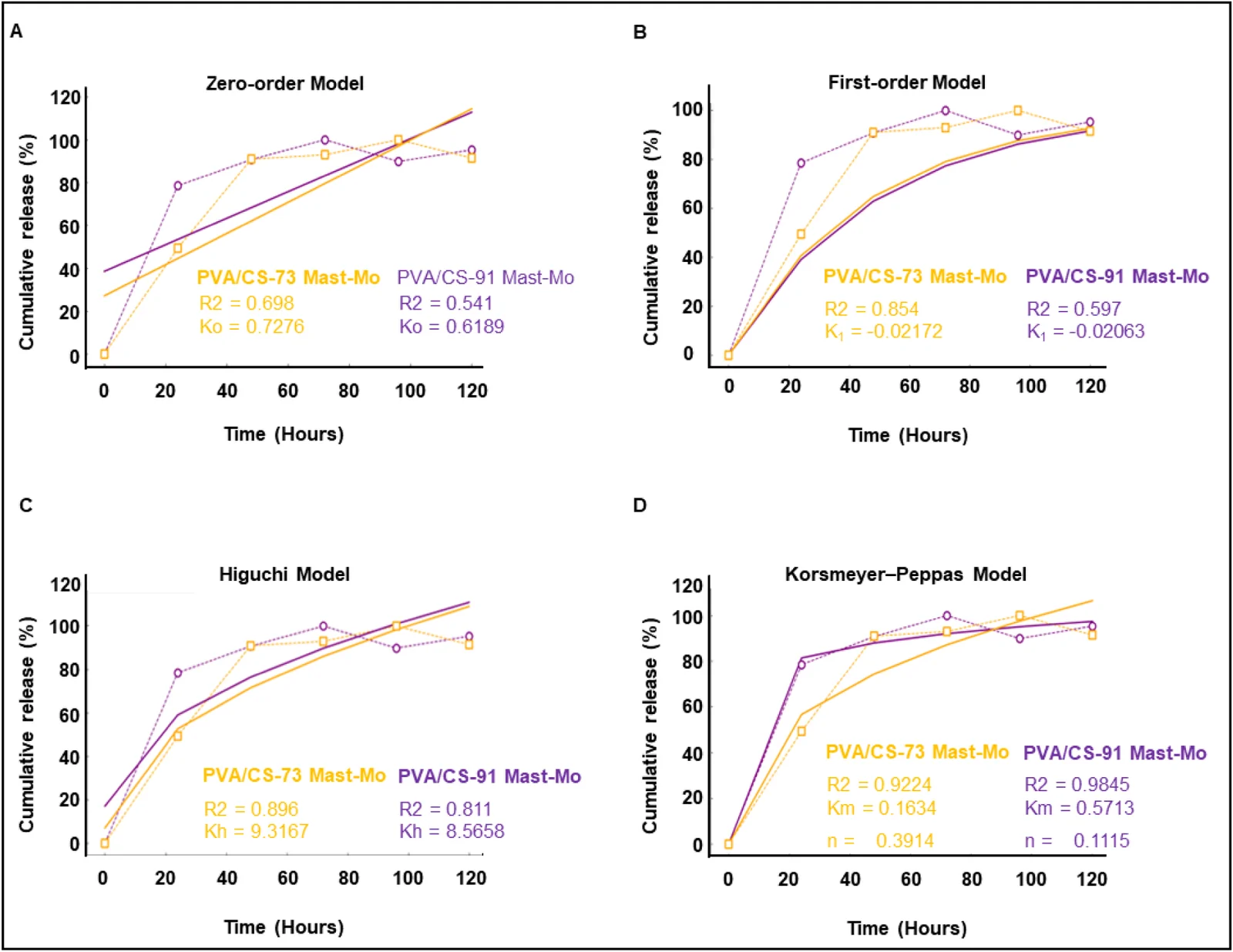
In vitro release behavior. The cumulative release (%) of the Mast-Mo peptide from PVA/CS-73 and PVA/CS-91 nanofiber wound dressings was measured at 24, 48, 72, 96, and 120 h. Release kinetics for PVA/CS-73 and PVA/CS-91 are presented using the following models: (A) zero-order, (B) first-order, (C) Higuchi, and (D) Korsmeyer–Peppas. PVA/CS-73 loaded with Mast-Mo is shown in orange, whereas PVA/CS-91 loaded with Mast-Mo is shown in purple. The corresponding kinetic parameters for each nanofiber mat are provided in the plots.

By contrast, PVA/CS-91 nanofiber wound dressings exhibited a more rapid release profile, with approximately 65% of the peptide released within the first 24 h (67.65 ± 3.08%) and 84.07 ± 12.41% released by 48 h. At 120 h, cumulative release increased to 94.14 ± 3.17%.

To describe the peptide release kinetics from the nanofiber wound dressings, four mathematical models were evaluated: zero-order, first-order, the Higuchi model, and the Korsmeyer–Peppas model (Figure 8A–D, respectively). The regression coefficients (R^2^) for each release model are shown in the graphs and summarized in Figure S2.

Notably, PVA/CS-73 nanofiber wound dressings exhibited release behavior consistent with a diffusion-controlled mechanism, as supported by the Higuchi model (*k*_h_ ≈ 9.31, R^2^ ≈ 0.896; Figure 8C). Similarly, analysis using the Korsmeyer–Peppas model indicated predominantly diffusion-controlled release from the polymeric matrix (n ≈ 0.39, R^2^ ≈ 0.922; Figure 8D).

In contrast, PVA/CS-91 exhibited a markedly more pronounced initial burst release than PVA/CS-73, complicating the interpretation of simple mechanistic models. Although the Higuchi analysis described the subsequent diffusional phase (*k*_h_ = 8.57, R^2^ = 0.811; Figure 8C), the excessive burst release within the first 24 h does not fully comply with the assumptions of the Higuchi model. Additionally, while the Korsmeyer–Peppas model provided a good overall fit (R^2^ = 0.9845; Figure 8D), the low diffusion exponent (n = 0.11) limits a straightforward mechanistic interpretation.

## Discussion

The use of electrospun nanofibers has emerged as a promising strategy for treating cutaneous wounds, offering advanced solutions that modulate the release of bioactive agents, prevent bacterial colonization, mimic the extracellular matrix, and create an optimal microenvironment for tissue regeneration. These features are crucial for enhancing the healing of complex wounds, including diabetic ulcers, partial-thickness burns, and extensive traumatic injuries.^14,19^

In this study, we developed electrospun nanofiber wound dressings that demonstrated significant antibacterial activity and the ability to stimulate cell proliferation and motility in primary human skin fibroblasts. Additionally, these wound dressings exhibited a significant capacity to accelerate the tissue regeneration process in an *ex vivo* human skin model, suggesting their potential as effective bioactive platforms for wound healing therapies.

The versatility and biocompatibility of PVA/CS based biomaterials make them well suited for wound-healing applications with antibacterial and pro-regenerative activity. Moreover, these materials exhibit favorable mechanical and physicochemical properties, essential for the development of effective wound dressings.^12,34^

Our results show that the average nanofiber diameter is influenced by the relative concentration of chitosan in the polymer solution, as well as by the presence of the bioactive peptide. CS concentrations are associated with fiber diameters,^35,36^ while the incorporation of peptides and polypeptides can modify solution properties such as surface tension and electrical conductivity, indicating that peptide addition can alter key factors governing the electrospinning process.^37,38^

FTIR analysis reveals that, in PVA/CS nanofiber, hydrogen bonding occurs between the hydroxyl groups of PVA and the amino groups of chitosan. This interaction is evidenced by a marked decrease in the intensity of the PVA acetate group band, together with shifts in the amide I (∼1650 cm^–1^) and amide II (1580–1550 cm^–1^) bands, confirming the formation of hydrogen bonds between the PVA and CS.^39,40^

Similarly, the incorporation of the Mast-Mo peptide (7.5 and 15 μM) into the nanofiber mats resulted in a shift of the absorption peak toward the ν­(O–H)/ν­(N–H) region, accompanied by a gradual decrease in transmittance, indicating the formation of new hydrogen bonds between the peptide functional groups (−NH_2_ and −COOH) and the PVA/CS matrix.^40,41^

The increase in peptide loading enhanced the intensity of the amide I band and decreased the transmittance of the amide II band, confirming the presence of supramolecular interactions. Moreover, the absence of new FTIR bands after peptide incorporation suggests that functionalization occurs through supramolecular physical interactions rather than covalent bonding, indicating that the peptide modulates fiber properties by reinforcing the hydrogen-bonding network.^41,42^

Overall, these findings indicate that the PVA/CS ratio directly affects interactions between the polymeric matrix and bioactive peptides, influencing hydrogen-bond density and the capacity to host supramolecular interactions. This is critical for designing nanofiber scaffolds tailored for biomedical applications such as drug delivery or wound healing.

Thermal analysis shows that Mast-Mo modulates the thermal properties of PVA/CS nanofiber mats in a polymer-ratio- and concentration-dependent manner. In PVA/CS-73 formulations, the progressive increase in ΔHf_1_ with increasing peptide concentration suggests enhanced retention of polar volatiles, likely driven by hydrogen-bond interactions between the peptide and both PVA/CS matrix.^43^ This effect may be amplified by the higher chitosan content, which could act synergistically with Mast-Mo through its protonated amino groups.^44^

Interestingly, in the PVA/CS-91 formulations, a nonmonotonic pattern suggests that the reduction in thermal stability is associated with low peptide concentrations, whereas higher peptide concentrations may induce polymer reorganization.^35^

Crystallinity analysis (ΔHf_2_) further supports this dual behavior. In PVA/CS-91 mats, crystallinity decreased at low peptide concentrations, likely due to disrupted polymer chain packing. In contrast, crystallinity increased at 15 μM, possibly reflecting peptide-mediated nucleation.^45^ Altogether, these findings indicate that Mast-Mo exerts a concentration-dependent and matrix-specific effect, being antagonistic at low concentrations in PVA-rich systems and synergistic at higher concentrations when chitosan is more abundant.^45^

Thermogravimetric analysis showed degradation behavior consistent with the previously reported three-stage profile for PVA/CS blend systems.^43^ The first stage corresponded to mass loss associated with the hydrophilic nature of PVA and chitosan.^43,44^ In contrast, the second stage can be attributed to PVA backbone dehydration and chain scission.^43,44^ At this stage, the higher T_onset_ observed in the PVA/CS-73 series compared to the PVA/CS-91 nanofiber wound dressings suggests a possible improvement in thermal stability. This behavior may be related to the higher chitosan content in the PVA/CS-73 formulations, which promotes additional intermolecular hydrogen bonding between the −OH and −NH_2_ groups of chitosan and the hydroxyl groups of PVA, thereby restricting segmental mobility and delaying the onset of degradation.^43,44,46^

In contrast, the third stage may be associated with the oxidative combustion of the carbonaceous char residue generated from chitosan.^44,46^ Interestingly, during this analysis, no peptide-specific thermal signal was detected in any TGA parameter. The peptide concentrations employed (13 and 26 μg) represent a negligible mass fraction relative to the polymer matrix, placing the expected thermal contribution below the detection threshold of conventional TGA instrumentation.^41^ Furthermore, peptide and protein degradation typically occurs between 200 and 350 °C, a range that completely overlaps with the dominant PVA degradation event, thereby preventing the resolution of a distinct peptide-related signal.^41^ Overall, these results confirm that peptide incorporation at the studied concentrations does not compromise the intrinsic thermal stability of the PVA/Cs nanofiber matrix.

In the design of new materials for wound treatment, the wettability of the dressing surface is a key factor in maintaining tissue moisture during the wound-healing process.^12,46^ Wettability plays a critical role in creating an environment that supports essential cellular behaviors, such as cell attachment, proliferation, and migration, while also facilitating the permeation of nutrients, metabolites, and oxygen to the tissue under repair.^47^

PVA and chitosan are inherently hydrophilic due to the presence of hydroxyl (−OH), amine (−NH_2_), and carboxyl (−COOH) groups, which promote strong interactions with water.^12,43^ The incorporation of bioactive molecules can modify wettability. The observed change in water contact angle upon Mast-Mo incorporation suggests an increased of hydrophilicity of the nanofiber wound dressings, likely driven by changes in surface chemistry and polymer–peptide interactions.^19,41^

In this context, all nanofibers, with or without the Mast-Mo peptide, exhibited adequate wettability, suggesting their potential to support tissue repair processes. Notably, all PVA/CS-73 nanofiber wound dressings showed greater wettability than those in the PVA/CS-91 group, a difference attributed to the higher chitosan content, which alters the degree of swelling.^46^

Complications associated with opportunistic infections remain a major challenge in the treatment of chronic nonhealing wounds.^2–4^ Our results demonstrate a significant reduction in bacterial viability after 24 h of treatment with Mast-Mo-loaded nanofiber wound dressings. This effect can be attributed to Mast-Mo ability to interact with and insert into bacterial outer membranes, where its intrinsic surface activity disrupts lipid packing, compromises membrane integrity, and ultimately leads to bacterial destabilization, a mechanism that has been demonstrated in vitro in several clinically relevant species, including *S. aureus*, *E. coli*, *S. pyogenes*, and *K. pneumoniae*.^26–28^ The antimicrobial effect against *P. aeruginosa* and *K. pneumoniae* was most pronounced in the nanofiber wound dressings containing the highest peptide concentration, which may be related to the antibacterial behavior of mastoparans, which shifts from surface interactions to more disruptive states as the peptide accumulates within the lipid bilayer, consistent with models in which local membrane concentration governs effective permeabilization.^26,48^

Likewise, the difference in sensitivity between Gram-positive bacteria (*S. aureus* and *S. epidermidis*) and Gram-negative bacteria (*P. aeruginosa* and *K. pneumoniae*) can be explained by structural differences in their cell envelopes. Antimicrobial peptides generally exhibit higher activity against Gram-positive bacteria due to the absence of an outer membrane, which facilitates direct access to the cytoplasmic membrane. In contrast, the outer membrane of Gram-negative bacteria, enriched in lipopolysaccharides, acts as a permeability barrier that limits peptide penetration and reduces antimicrobial efficacy.^49,50^

In addition to the antimicrobial activity of Mast-Mo, chitosan itself has been reported to exhibit intrinsic antibacterial properties due to its polycationic nature, which promotes electrostatic interactions with negatively charged bacterial surfaces and alters membrane permeability. This effect is often more pronounced in Gram-positive bacteria, likely due to the absence of an outer membrane, which facilitates direct access to the cell wall. However, the antibacterial activity of chitosan is highly dependent on several factors, such as molecular weight, degree of deacetylation, pH, and bacterial strain. This feature may account for the reduction in bacterial viability observed in both nanofiber mats, even in the absence of the peptide.^51,52^

In this study, the nanofiber wound dressings loaded with the Mast-Mo peptide exhibited broad biocompatibility, which can be attributed to two main factors. First, the polymers selected for scaffold fabrication. Poly­(vinyl alcohol) (PVA) and chitosan (CS) have been widely reported as promising platforms for the controlled release of bioactive substances, minimizing cytotoxicity and preventing tissue damage.^11–14^ Second, the use of a highly biocompatible bioactive molecule, such as the Mast-Mo peptide, which shows low cytotoxicity across several cell models (HEK293, L929, and RAW 264.7), represents a critical requirement for the development of safe and effective therapeutic strategies.^28,29^

Skin wound repair requires coordinated cell proliferation and migration to replace damaged cells and restore the integrity of the skin barrier.^3,4^ Nanofiber wound dressings loaded with the Mast-Mo peptide significantly enhanced cell proliferation and migration under in vitro conditions, suggesting their potential as a promising therapeutic strategy for chronic and difficult-to-heal wounds.

This effect may primarily be attributed to the pro-regenerative properties of the Mast-Mo peptide, which has been shown to induce cell proliferation, migration, and the expression of pro-repair genes in skin tissue models.^29^ In addition, the pro-regenerative potential of these nanofiber mats may also be associated with the presence of chitosan (CS) in the nanofiber structure. This biopolymer has been reported to stimulate the expression of growth factors, pro- and anti-inflammatory cytokines, chemokines, and bioactive lipids, all of which support skin wound healing by enhancing cell–material interactions and promoting cell migration and proliferation processes essential for tissue repair.^15,16^

Several nanofiber systems incorporating antimicrobial peptides have demonstrated the ability to accelerate wound healing and prevent biofilm formation in several *in vivo* models, including diabetic and chronic wound settings.^53–55^ For example, poly­(ethylene oxide)/poly­(d,l-lactide) nanofibers loaded with nisin accelerate wound healing in mice,^53^ silk fibroin nanofibers containing HPRP-A2 improve wound closure in diabetic models,^54^ and core–shell nanofibers based on Pluronic F127 and PCL loaded with the cathelicidin peptide 17BIPHE2 effectively prevent bacterial biofilm formation and promote chronic wound repair.^55^ These findings suggest that nanofiber-based drug delivery formulations involving bioactive molecules represent ideal candidates for a wide range of applications, particularly in wound dressings, orthopedic implant-related infections, antibacterial treatment of biofilms, and tissue-engineered scaffolds.^56^

Although few commercial products based on electrospun nanofibers are currently available on the market, reflecting a significant translational gap, electrospinning-based technologies such as Restrata (Registered Trademark), Phoenix Wound Matrix (Registered Trademark), and Spin-Care have been developed and evaluated in clinical settings. For example, SpinCare, which enables the *in situ* deposition of nanofibers directly onto wounds, forming conformal, breathable barriers that promote re-epithelialization, has been evaluated in clinical pilot trials and has demonstrated improved healing outcomes, reduced pain, and enhanced patient comfort compared to conventional wound care.^57,58^ Similarly, the evaluation of a fully synthetic electrospun fiber matrix (Restrata) for the treatment of post-traumatic soft tissue defects, showed that this type of wound dressing, combined with negative pressure therapy, promoted granulation tissue formation and supported wound healing, with most patients achieving complete recovery and minimal complications after a single application.^59^

These findings strongly suggest that biomimetic nanofiber structures can promote tissue regeneration through a noninvasive application, offering practical advantages in clinical settings. In contrast to conventional products that lacks a high degree of biomimicry, nanofiber-based wound dressings may provide enhanced support for angiogenesis, antimicrobial activity, and overall healing outcomes in wound management, thereby highlighting their excellent biocompatibility, structural mimicry, and capacity to improve clinical outcomes in advanced wound healing applications.^57,58^

Several studies have investigated PVA/CS nanofiber-based wound dressings in both *in vitro* and *in vivo* models, demonstrating their antimicrobial capacity and ability to enhance wound healing. In particular, the incorporation of bioactive molecules from several origins has led to the development of materials with broad-spectrum antibacterial activity against clinically relevant microorganisms, supporting their potential application in skin regeneration across various contexts, including diabetic, burn, and infected wounds.^60–62^

Despite these advances, a significant proportion of these studies have been conducted in conventional *in vivo* models, which present important translational limitations. Although widely used, various animal models often fail to fully replicate human skin physiology, immune responses, and wound healing dynamics. Differences in skin structure, healing rates, and microbiome composition, among other factors, may limit the predictive value of these findings. Moreover, controlled experimental conditions may not accurately reflect the complexity of clinical environments, restricting the direct applicability of results to human patients.^63^

Considering this, the present study proposes the use of alternative models based on *ex vivo* human skin cultures derived from healthy skin explants, which preserve the native tissue architecture and cellular heterogeneity. These types of models have emerged as a viable option with high translational potential, as they provide a physiologically relevant platform to investigate metabolic activity, cell–cell interactions, and tissue responses under conditions that closely resemble human skin *in vivo.* ^31,32^

They also enable evaluation of key wound healing processes, including re-epithelialization, inflammatory responses, and extracellular matrix remodeling, under controlled yet biologically relevant conditions, reducing reliance on animal models while offering both experimental and ethical advantages.^31,33^ Importantly, such human tissue-derived platforms are well-suited for evaluating advanced biomaterials, including electrospun nanofiber wound dressings. Consequently, integrating biomimetic biomaterials systems with *ex vivo* human skin models represents a significant advance, providing a clinically relevant framework that helps bridge the translational gap by improving the predictive value of preclinical data while minimizing interspecies variability.^64^

Within this framework, our experimental observations demonstrate a significant reduction in lesion area following treatment of the hOSEC model with nanofiber wound dressings loaded with Mast-Mo, indicating pro-regenerative effects in real human skin model. This response may be associated with the intrinsic bioactivity of the Mast-Mo peptide. In *ex vivo* human skin lesion models comparable to the one used in this study, this peptide has been shown to accelerate tissue repair and upregulate the expression of KI67, HAS2, and CXCR4, genes closely associated with cell proliferation, extracellular matrix remodeling, and cell migration during wound healing.^29^

It has been suggested that Mast-Mo and its analogs can promote leukocyte recruitment to the site of infection and reduce the expression of pro-inflammatory cytokines such as IL-12, TNF-α, and IL-6,^28,48^ while also facilitating mast cell modulation and regulating TNF-α and IL-1β expression.^25^ Together, these effects represent key properties that support tissue regeneration, control infection, and modulate excessive inflammatory responses.^25,28,48^

Interestingly, this potential immunomodulatory property of the biomaterial, derived from the presence of the Mast-Mo peptide, may be key to tissue regeneration and the management of opportunistic infections, offering an immunomodulatory biomaterials-based strategy for the treatment of chronic wounds.^65,66^

Nanofiber systems enable high drug loading and controlled, targeted release, improving the stability and delivery of peptides/proteins and growth factors across diverse therapeutic applications, such as the treatment of chronic wounds.^18^

Release kinetics analysis shows that PVA/CS-73 nanofibers exhibit a more controlled release behavior, consistent with a diffusion-based mechanism. The Higuchi model (Kh ≈ 9.32, R^2^ ≈ 0.896), first-order model (R^2^ ≈ 0.855), and Korsmeyer–Peppas model (n ≈ 0.39, R^2^ ≈ 0.922) consistently support the conclusion that the release behavior of PVA/CS-73 nanofiber wound dressings is predominantly diffusion-controlled.^20,62^

In contrast, PVA/CS-91 nanofiber mats exhibit a markedly pronounced burst release phase, indicating deviation from simple mechanistic models. The first-order model shows a poor fit (R^2^ ≈ 0.597), whereas the Korsmeyer–Peppas model provides a good fit (R^2^ ≈ 0.985) but yields a low diffusional exponent (n ≈ 0.11), rendering the model mechanistically uninterpretable. Nevertheless, this parameter remains useful for describing the release behavior, suggesting a higher drug concentration at or near the nanofiber surface in this formulation.^18^

Similar release profiles to those observed for PVA/CS-73 nanofiber wound dressings have been reported in nanofiber systems with comparable characteristics, in which bioactive compounds such as curcumin, ofloxacin, or fish-derived antioxidant peptides (AOPs) display an initial burst release followed by a slower, sustained release phase.^19,41,67^ The initial burst is commonly attributed to the dissolution of drug molecules located on or near the nanofiber surface or to rapid matrix swelling, which promotes the formation of pores and channels within the fiber structure.^18^ Subsequently, the controlled release phase is governed by the slow diffusion of the bioactive peptide from within the polymer matrix or by gradual material erosion, enabling Mast-Mo release from deeper regions of the nanofibers.^19,20,62,67^

## Methods

### Materials

Poly­(vinyl alcohol) (PVA, Mw: 85,000–124,000, 87–89% hydrolyzed) was purchased from Dinâmica Química Contemporânea Ltd. (São Paulo, SP, Brazil). Chitosan (CS, low molecular weight, degree of deacetylation 75–85%), Glacial acetic acid, penicillin/streptomycin solution, dimethyl sulfoxide (DMSO), and MTT assay kit were provided by Sigma Chemicals Co. (St. Louis, MO, USA). Dulbecco’s Modified Eagle’s Medium (DMEM) and fetal bovine serum (FBS) were obtained from Gibco (Waltham, MA, USA). Mueller-Hinton agar and Tryptic Soy Broth (TSB) medium were provided by Kasvi Ltd. (São José dos Pinhais, PR, Brazil). Mastoparan-Mo peptide (FLPIIINLKALAAALAKKIL)^28^ was synthesized and supplied by Peptide 2.0 Inc. (Chantilly, VA, USA).

### Preparation of Solvent and Nanofiber Wound Dressings Fabrication

A PVA solution (10% w/v) was prepared in ultrapure water at 85 °C under constant stirring for 4 h. A CS solution (3% w/v) was dissolved in acetic acid (10% v/v) under continuous stirring at 60 °C for 3 h. The PVA and CS solutions were combined in two volumetric ratios: 7:3 (PVA/CS-73) and 9:1 (PVA/CS-91). The mixtures were stirred under magnetic conditions at ambient temperature to form a stable suspension. The Mast-Mo peptide was solubilized in 0.1% acetic acid and added to the respective PVA/CS mixtures under constant stirring for 30 min in two different concentrations (7.5 and 15 μM). The PVA/CS mixtures, with and without the Mast-Mo peptide, were then electrospun using a Nano Fiber Electrospinning System (CY Scientific Instrument Co., China) coupled to an automatic syringe pump (New Era Pump System Inc., Farmingdale, NY, USA). The solution was loaded into a 20 mL syringe connected to a stainless steel 21-gauge blunt-ended needle at room temperature. The electrospinning parameters were set as follows: solution discharge rate of 0.5 mL/h, applied voltage of 16 kV, and a distance of 15 cm between the needle tip and the aluminum-foil-covered collector. To ensure fiber concentration on the collector surface, other parts of the machine were insulated, except for the section where the nanofibers were deposited onto the aluminum foil.

### Scanning Electron Microscopy (SEM)

The ultrastructure of the nanofibers was evaluated using scanning electron microscopy (SEM) with a TESCAN MIRA fourth generation scanning electron microscope (TESCAN, Brno, Czech Republic), operating at an accelerating voltage of 10 kV. All scaffolds were gold-coated prior to analysis using a Denton Vacuum Desk V sputter coater (Denton Vacuum, Moorestown, NJ, USA) for 120 s at 30 mA. The average diameter of the fabricated nanofibers was determined using ImageJ software (National Institutes of Health, Bethesda, MD, USA). A total of 50 nanofibers were measured across 10 randomly selected fields at 10,000× magnification. Morphometric analysis was conducted using three independent nanofiber wound dressing samples for each material.

### Fourier Transform Infrared Spectroscopy (FTIR)

Functional groups of fabricated nanofibers were evaluated using Fourier Transform Infrared Spectroscopy (FTIR). The FTIR spectroscopy was performed on a Frontier FT-IR/FIR instrument (PerkinElmer, Waltham, Massachusetts, U.S.), equipped with an attenuated total reflectance (ATR) accessory. The analysis was performed in the 4000–600 cm^–1^ region, with 60 scans and a resolution of 4 cm^–1^.

### Thermal Behavior Using DSC

The thermal behavior was evaluated using differential scanning calorimetry (DSC) on a Hitachi DSC 7020 Thermal Analysis System (Hitachi, Chiyoda, Tokyo, Japan). For the analysis, the samples (10 ± 3 mg each) were subjected to two heating cycles and one cooling cycle, each carried out at 10 °C/min under a nitrogen atmosphere with a flow rate of 50 mL/min. The analysis was conducted from 0 to 220 °C (first heating cycle), then cooled to −20 °C (cooling cycle), and finally heated from −20 to 200 °C (second heating cycle).

### Thermogravimetric Analysis (TGA)

TGA analyses were performed on a Tarsus TGA 209 F3 (NETZSCH, Germany) to determine the percentage of extractable material and the material’s thermal stability. Temperature ranged from 25 to 700 °C under a nitrogen atmosphere (20 mL/min) and a heating rate of 10 °C min^–1^ using platinum crucibles. The gas purge flow was maintained at 70 mL/min.

### Swelling and Degradability of Nanofiber Scaffolds

To evaluate changes in swelling rate, the material was cut into 1 × 1 cm squares. The dry weight (Wdt) of each material was recorded, and then it was submerged in PBS (pH 7.4) at 37 °C for 24 h to evaluate water uptake. The samples were washed with deionized water and then weighed after the excess water was removed. The degree of swelling or water absorption was calculated according to Fathi et al. (2020),^12^ using eq 1.

1 Swellingratio(%)=(Wst−Wdt)/Wdt×100

where Wst is the wet weight after immersion in PBS.

To evaluate the degradability of the nanofiber mats, squares of 2 × 2 cm of each material were immersed in PBS and incubated at 37 °C for 2, 7, and 14 days. The samples were removed from the buffer solution and washed with deionized water at room temperature. Subsequently, the fibers were gently placed between two filter papers to remove excess water, and the final material weight (fW) was determined. The weight-loss percentage (Wl%) was calculated using eq 2.

2 Wl(%)=(Wf−Ws)/Ws×100

### Contact Angle Determination

Surface wettability was evaluated by measuring the water contact angle using a goniometer (Attension Theta Lite, Biolin Scientific, Finland). Electrospun nanofiber mats were fixed onto glass microscope slides using double-sided adhesive tape to ensure a flat and stable surface. A droplet of deionized water (2 μL) was gently deposited onto the sample surface under ambient conditions (25 °C and 35 ± 5% relative humidity). The contact angle was recorded within 10 s after droplet deposition using image analysis software. For each sample, measurements were performed at five different positions to account for surface heterogeneity. The reported values correspond to the mean ± standard deviation of three independent experiments (n = 3). Group comparisons were performed using one-way ANOVA, with differences considered statistically significant at *p* ≤ 0.05.

### Peptide Release Analysis

The release behavior of the nanofiber wound dressings was characterized using an in vitro elution method, as described in previous reports.^19,68,69^ Briefly, electrospun membranes (10 mg/mL) were immersed in PBS (pH 7.4) and incubated at 37 °C for 24, 48, 72, 96, and 120 h. At each time interval, 100 μL of the eluent was removed and evaluated. Fresh phosphate-buffered solution (100 μL) was added after each sampling to maintain a constant volume. The optical density of the samples was measured at 205 nm using a NanoDrop One microvolume UV–Vis Spectrophotometer (Thermo Fisher Scientific, Waltham, MA, USA). The results represent the mean of three independent experiments. To describe the peptide release kinetics, the data were fitted to four mathematical models^68^ (Table S3; eqs S1–S4).

### Antibacterial Activity

The antibacterial activity of electrospun wound dressings was evaluated by determining the number of colony-forming units (CFU) on a plate following interaction with the nanofiber mats according to CLSI guidelines (CLSI M07-A11, 2018).^70^ Fresh colonies of *Staphylococcus aureus* (ATCC 25923), *Klebsiella pneumoniae* (5121467 LACEN-DF), *Staphylococcus epidermidis* (ATCC 12228), and *Pseudomonas aeruginosa* (ATCC 27853) were inoculated in tryptic soy broth (TSB) medium and incubated for 16 h at 37 °C. Bacterial growth was monitored until the absorbance at 600 nm reached 0.6 OD (4 × 10^8^ CFU/mL), corresponding to the logarithmic growth phase. The bacterial suspension was then diluted to a final concentration of 1 × 10^5^ CFU/mL. The nanofiber wound dressings (2.5 mg/mL) were immersed in the bacterial suspension and incubated at 37 °C for 24 h under aerobic conditions. Ciprofloxacin (40 μg/mL) was used as a positive control for antibacterial activity (Crt +), while TSB medium served as the negative control (Crt −). After incubation, the bacterial suspension was diluted and inoculated onto Mueller-Hinton agar plates, which were incubated at 37 °C for 24 h. The number of colonies was subsequently counted. Results were expressed as the mean ± standard deviation of the bacterial viability percentage. Each experiment was performed in triplicate, and group comparisons were conducted using Kruskal–Wallis test followed by Dunn’s test with Bonferroni correction versus control (Crt, −). Differences with *p* ≤ 0.05 were considered statistically significant.

### Cell Lines and Maintenance

hFibs cells were isolated from skin fragments obtained from healthy donors undergoing abdominoplasty procedures performed at Clínica Carpaneda Cirurgia Plástica (Brasília, Brazil), following approval by the Research Ethics Committee of the University of Brasília (Protocol No. 0175020.0.0000.5558) and after obtaining informed consent. After removal of the hypodermis, the tissue was minced into small fragments and incubated overnight at 4 °C with Dispase solution (4 mg/mL). The dermis was then mechanically separated from the epidermis, and the dermis was digested with type II collagenase (1 mg/mL) for 3 h. Collagenase activity was neutralized with DMEM supplemented with 10% fetal bovine serum (FBS).

The cell suspension was centrifuged at 2,000 rpm for 10 min, the supernatant discarded, and the cell pellet resuspended in DMEM containing 10% (v/v) FBS. Cells were cultured in DMEM supplemented with 10% (v/v) FBS and 1000 U/mL penicillin/streptomycin solution, and maintained at 37 °C, 5% CO_2_, and 95% humidity.

### Cytotoxicity Tests

The nanofiber wound dressings were sterilized under UV light for 4 h on each side and stored at 4 °C until use. Cytotoxicity assays were conducted in accordance with the ISO 10993-5 standard test method.^71^

The scaffolds were incubated in DMEM supplemented with 10% (v/v) FBS for 24 h at 37 °C, using an extraction ratio of 10 mg of electrospun membrane per mL of medium. DMEM containing 25% methanol was used as the positive control for toxicity, while DMEM with 10% FBS served as the negative control. hFibs cells were seeded at a density of 1 × 10^4^ cells per well in 96-well plates and incubated under standard conditions (5% CO_2_, 37 °C, 95% humidity). After 24 h, the culture medium was replaced with the nanofiber extract, and the cells were exposed for an additional 24 h. Cytotoxicity was evaluated using the MTT assay. Following incubation, the nanofiber extracts were removed, and the wells were washed with 200 μL of PBS followed by 200 μL of DMEM. Then, 200 μL of thiazolyl blue tetrazolium bromide (MTT) solution in DMEM (0.5 mg/mL) was added to each well, and the plates were incubated in the dark at 37 °C for 4 h. The MTT solution was subsequently removed, and 200 μL of dimethyl sulfoxide (DMSO) was added to each well. The plates were then incubated in the dark with constant agitation for 30 min. Absorbance values were measured using a microplate reader (Bio-Tec PowerWave HT, USA) at 595 nm with a reference wavelength of 650 nm. The results were expressed as the mean ± standard deviation of cell viability percentage relative to the control group. Group comparisons were performed using one-way ANOVA analysis, with differences considered statistically significant at *p* ≤ 0.05.

### Cell Proliferation and Cell Migration Assay

hFibs cells were seeded in 24-well plates at a density of 2 × 10^4^ cells per well and incubated in DMEM supplemented with 10% (v/v) FBS at standard conditions. After 24 h of incubation, the culture was washed with serum-free medium, and the medium was replaced with fresh medium. The cells were treated with 1 mg/mL of the electrospun wound dressings in DMEM supplemented with 1% FBS. DMEM supplemented with 10% FBS was used as the positive control for proliferation, while medium supplemented with 1% FBS served as the negative control. Medium supplemented with 1% FBS was used as a low-serum condition to reduce basal proliferation while preserving cell viability, enabling a controlled baseline for comparison, whereas 10% FBS was used as a positive control to provide optimal growth conditions and maximal proliferation, allowing assessment of the intrinsic effects of the electrospun nanofiber wound dressings on cell proliferation.

The cells were incubated for 2, 4, and 7 days in the presence of the nanofiber wound dressings, with the medium volume maintained by adding fresh medium every 2 days. At the end of the incubation period, the nanofiber scaffold and culture medium were removed, and the wells were washed with 200 μL of PBS followed by 200 μL of DMEM. Subsequently, 200 μL of thiazolyl blue tetrazolium bromide (MTT) solution in DMEM (0.5 mg/mL) was added, and the MTT assay was performed as described previously. The results were expressed as the mean ± standard deviation of the proliferation percentage relative to the positive control (100%). Statistical comparisons between groups were conducted using one-way ANOVA, with differences considered significant at *p* ≤ 0.05.

The effect of nanofiber wound dressings on the migratory capacity of hFibs cells was assessed using the wound scratch assay, as described by Liang et al. (2007).^72^ Briefly, hFibs were seeded in 6-well plates and cultured until confluence. The culture was washed with serum-free medium, and the medium was replaced with fresh medium. Scratches were then introduced into the cell monolayers using 200 μL pipet tips. Cell debris was removed by washing with PBS, followed by the addition of a fresh culture medium. The cells were treated with 1 mg/mL nanofiber in DMEM supplemented with 1% FBS, which served as the vehicle. DMEM supplemented with 10% FBS was used as the positive migration control, while the vehicle alone served as the negative control. The cultures were incubated at 37 °C and imaged at 0 and 24 h using a Zeiss Primo Vert microscope equipped with a digital camera (Carl Zeiss, Heidelberg, Germany). The area invaded by the initial scratch was quantified using ImageJ software (National Institutes of Health, USA). Cell migration was quantified using the area method, according to Bobadilla et al. (2019).^73^ Wound closure was evaluated by measuring the relative wound area over time, as described in eq 3, where A_24_ represents the wound area at 24 h and A_0_ corresponds to the initial wound area. Statistical comparisons between groups were performed using one-way ANOVA. Differences were considered significant at *p* ≤ 0.05.

3 $$\mathrm{Wound area}=\left(A_{24}/A_{0}\right)\times 100$$

### Wound Healing Assay in an Ex Vivo Human hOSEC Model

Human skin samples were obtained as surgical discards from abdominoplasty procedures performed at Clínica Carpaneda Cirurgia Plástica (Brasília, Brazil) as described above. All donors provided written informed consent before participation, and the study protocol was approved by the Ethics Committee of the Faculty of Medicine, University of Brasília (protocol no. 87027125.6.0000.5558). Ex vivo organotypic human skin cultures were established, and a wound-healing assay was carried out as described by Alencar-Silva et al. (2024).^29^ Briefly, skin explants were washed with PBS containing penicillin/streptomycin, the hypodermis was removed, and 6 mm full-thickness punch biopsies were obtained. Explants were placed in metallic supports under air–liquid interface conditions at 37 °C, 5% CO_2_, and 95% humidity in DMEM supplemented with 10% FBS and penicillin/streptomycin. Circular wounds (2.5 mm) were created, rinsed with PBS, and explants were randomly allocated into five groups (n = 3): (1) DMEM without FBS (control), (2) PVA/CS-73 with 0 μM Mast-Mo, (3) PVA/CS-73 with 15 μM Mast-Mo, (4) PVA/CS-91 with 0 μM Mast-Mo, and (5) PVA/CS-91 with 15 μM Mast-Mo. Treatments with nanofiber wound dressings were applied immediately after wounding. Wound healing was evaluated after 7 days by measuring changes in wound area using ImageJ software (National Institutes of Health, USA), and statistical analysis was performed by one-way ANOVA (*p* ≤ 0.05).

### Statistical Analysis

All experiments were conducted in three or more biologically independent replicates, in addition to technical replicates, as detailed in each assay. Data were analyzed using GraphPad Prism software, version 7.02 (GraphPad Software, LLC, San Diego, CA, USA, 2017). FTIR and DSC data were analyzed using OriginPro software, version 2023 (OriginLab Corporation, Northampton, MA, USA, 2023). In all experiments, a p-value of less than 0.05 was considered significant.

## Conclusions

In the present study, a series of bioactive nanofiber wound dressings incorporating the antimicrobial and pro-regenerative peptide Mast-Mo were developed and evaluated for their antibacterial activity and potential in wound treatment, both *in vitro* and in an *ex vivo* human skin model.

These nanofiber mats exhibited well-defined ultrastructural organization, suitable physicochemical properties, high hydrophilicity, and controlled release behavior, supporting their potential as functional biomaterials. Likewise, these biomaterials exhibited potent, broad-spectrum antibacterial activity against clinically relevant pathogens, including *S. aureus*, *P. aeruginosa*, *K. pneumoniae*, and *S. epidermidis*, suggesting their strong potential to effectively prevent, control, and reduce infections commonly associated with acute and chronic skin wounds.

Moreover, *in vitro* evaluations demonstrated that the developed nanofiber mats are highly biocompatible, even at elevated concentrations, and actively promote human fibroblast proliferation and migration, thereby enhancing in vitro wound closure. Importantly, *ex vivo* validation using a human skin explant model demonstrated that these nanofiber dressings actively promote tissue regeneration in a clinically relevant human skin environment, highlighting their translational potential and supporting progression toward preclinical and clinical wound healing applications.

Overall, the Mast-Mo-loaded nanofiber wound dressings, combining appropriate nanofibrous architecture with potent antimicrobial activity and enhanced pro-regenerative effects, represent a promising multifunctional platform for advanced wound healing applications. Future work should extend these findings through *in vivo* validation and mechanistic studies addressing immunomodulatory effects and signaling pathways governing proliferation, migration, and tissue regeneration.

## Supplementary Material



## Data Availability

The authors declare that the data supporting the findings of this study are available within the paper and its Supporting Information files. Data are available from the corresponding author upon request.

## Notes

Figure S1: nanofiber diameter analysis; diameter distribution of nanofiber wound dressings: (A) PVA/CS-73 and (B) PVA/CS-91 formulations containing different peptide concentrations; statistical analysis using one-way ANOVA; significant differences indicated by asterisks (*** *p* < 0.001); Figure S2: cumulative release behavior and kinetic parameters; Figure A: cumulative release (%) of the Mast-Mo peptide from PVA/CS-73 and PVA/CS-91 nanofiber wound dressings at 24, 48, 72, 96, and 120 h; Figure B: corresponding kinetic parameters for the release models (zero-order, first-order, Higuchi, and Korsmeyer–Peppas); Table S1: characteristic temperatures (T) and enthalpy values (ΔH) of the thermal transitions observed in PVA/CS Mast-Mo nanofiber mats; Table S2: thermogravimetric analysis (TGA) parameters of PVA/CS electrospun membranes loaded with Mast-Mo peptide at different concentrations; Table S3: mathematical models used to describe peptide release kinetics from PVA/CS Mast-Mo nanofiber mats (PDF)

## Abbreviation

ECM: extracellular matrix
PVA: poly­(vinyl alcohol)
CS: chitosan
FDA: Food and Drug Administration
AMPs: antimicrobial peptides
Mast-Mo: Mastoparan-Mo
TNF-α: tumor necrosis factor alpha
IL-6: interleukin-6
hOSEC: human Organotypic Model of Human Skin
SEM: scanning electron microscopy
FTIR: Fourier Transform Infrared Spectroscopy
ATR: attenuated total reflectance
DSC: differential scanning calorimetry
hFibs: primary human dermal fibroblasts
PBS: phosphate-buffered saline
DMSO: dimethyl sulfoxide
MTT: MTT assay (thiazolyl blue tetrazolium bromide)
DMEM: Dulbecco’s Modified Eagle’s Medium
FBS: fetal bovine serum
TSB: Tryptic Soy Broth
CFU: colony-forming units
CLSI: Clinical and Laboratory Standards Institute
ATCC: American Type Culture Collection
ISO: International Organization for Standardization
UV: ultraviolet light
OD: optical density
CO_2_: carbon dioxide
rpm: revolutions per minute
mL: milliliter
μL: microliter
mg: milligram
μg: microgram
nm: nanometer
μm: micrometer
kV: kilovolt
mA: milliampere
cm^–1^: wavenumber
°C: degrees Celsius
kH: Higuchi model constant
R^2^: coefficient of determination
ΔHf_1_/ΔHf_2_: enthalpy of fusion (components 1 and 2)
