# Supplementary material for: Skin Wound Dressings Based on Biocompatible Nanofibers Loaded with the Mastoparan-Mo Peptide to Promote Regeneration and Control Infection in Skin Wounds
Source: ACS Omega. 2026 Oct 5;11(41):62544–63. doi: 10.1021/acsomega.6c02591 (PMC13689217; doi:10.1021/acsomega.6c02591)
Supplement: Supplementary file 1 [file ao6c02591_si_001.pdf]

Supporting Information

Skin Wound Dressings Based on Biocompatible Nanofibers Loaded with the Mastoparan-Mo Peptide to Promote Regeneration and Control Infection in Skin Wounds.

Rubén D. Díaz-Martin[1*], Angela Y. Becerra-Lovera[2], Guilherme Varejão S. Pasqual[1], Nathália Ribeiro Lobo[1], Stefhani Barcelos[1,3], Maria Victória Souto-Silva[3], Juliana Lott de Carvalho[3], Octávio Luiz Franco[1, 4], Marcos L. Dias[2], Simoni Campos Dias [1.]

1. Programa de Pós-graduação em Ciências Genômicas e Biotecnologia, Universidade Católica de Brasília, Brasília, DF, Brazil, PC: (CEP) 71966-700 Brasília, DF, Brazil
2. Instituto de Macromoléculas Professora Eloisa Mano, Universidade Federal do Rio de Janeiro, Rio de Janeiro, Brazil, PC: (CEP) 21941-598 – Rio de Janeiro, RJ, Brazil
3. Faculdade de Medicina, Universidade de Brasília, Brasília, DF, Brazil, PC: (CEP) 70910-900 Brasília, DF, Brazil
4. S-Inova Biotech, Programa de Pós-Graduação em Biotecnologia, Universidade Católica Dom Bosco, PC: (CEP) 79117-010 Campo Grande, MS, Brazil

Corresponding authors: *Rubén D. Díaz-Martin (rubendario025@gmail.com)*

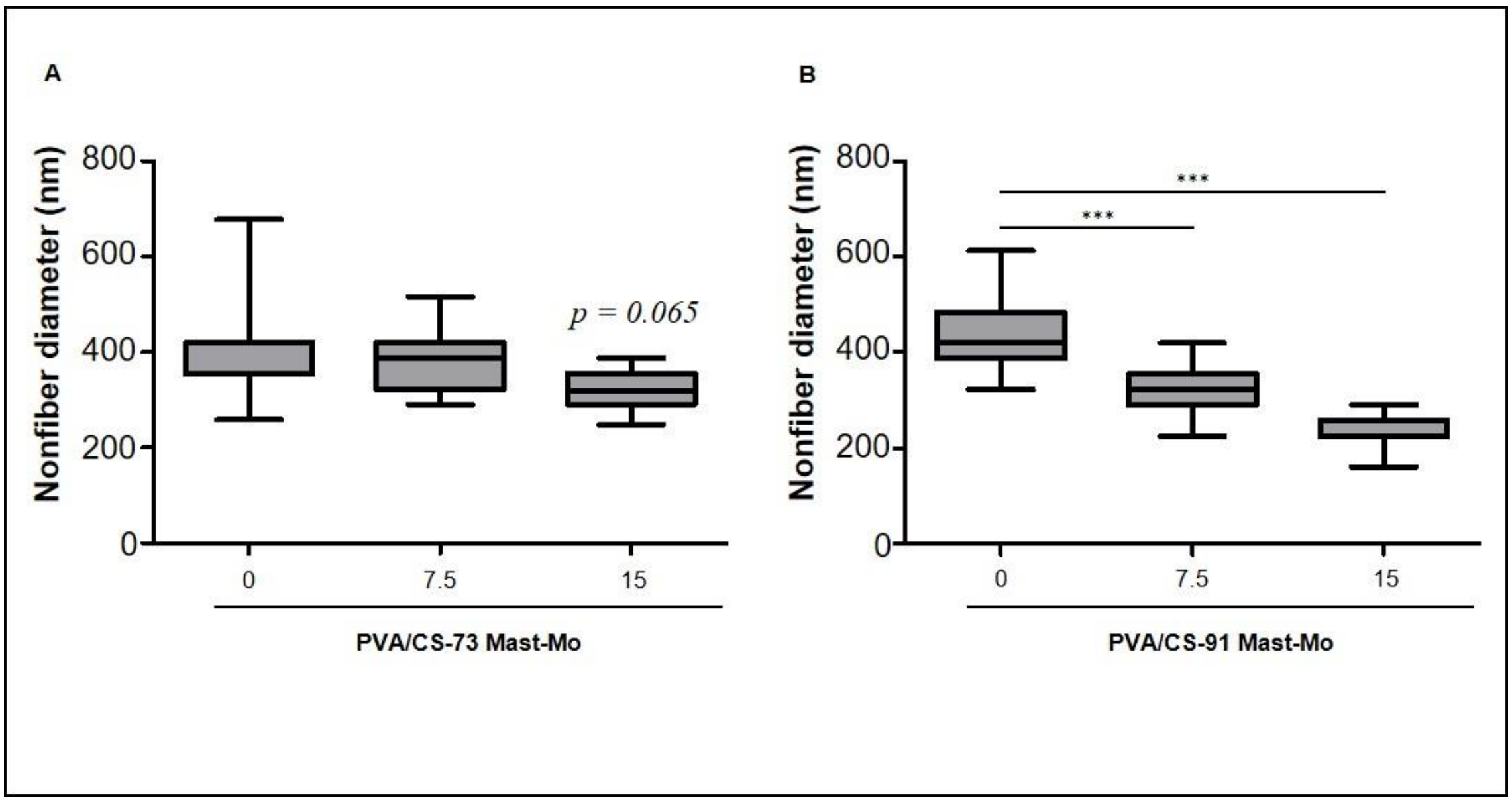

Figure S1. Nanofiber diameter analysis. Diameter distribution of nanofiber wound dressings (A) PVA/CS-73 and (B) PVA/CS-91 formulations containing different peptide concentrations. Statistical analysis was performed using one-way ANOVA, and significant differences are indicated by asterisks (*** $p < 0.001$).

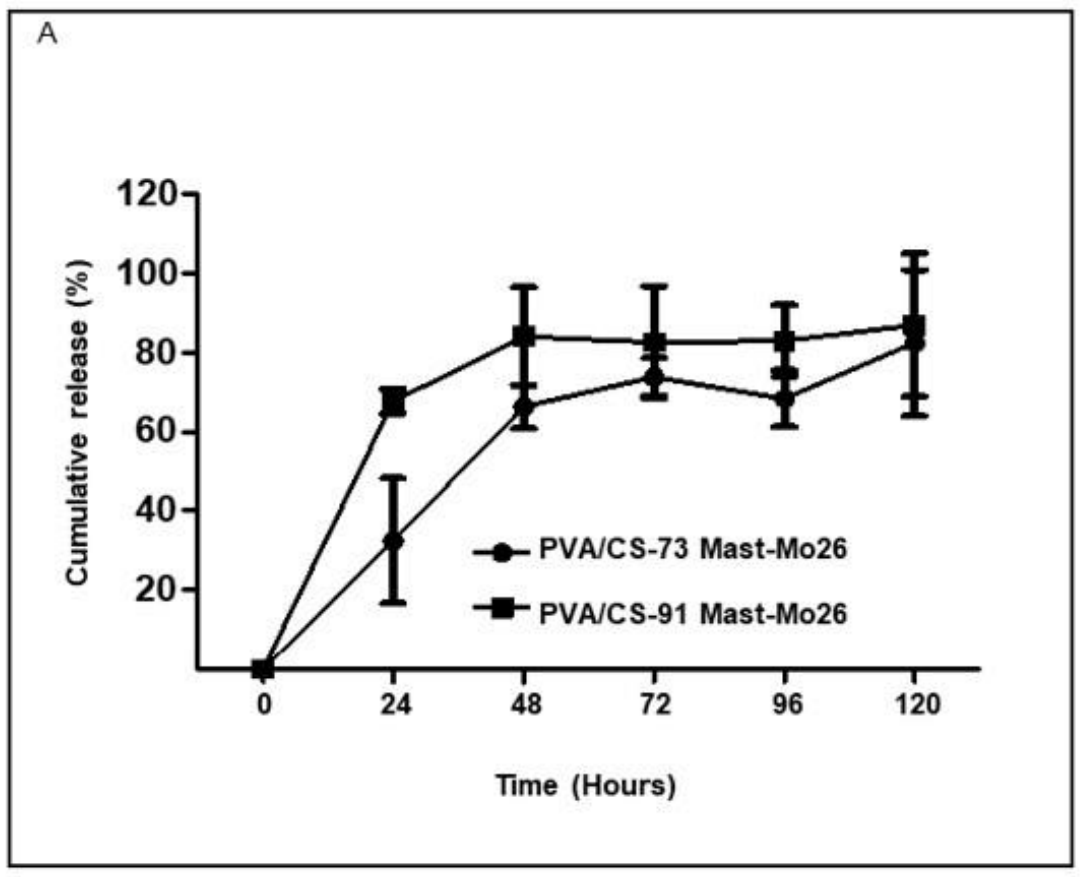

B

Models and parameters	*Kinetic parameters of PVA/CS nanofiber mats*	
	**PVA/CS-73**	**PVA/CS-91**
**Zero-order**		
Equation	y = 0.7276x + 27.1370	Y = 0.6189x + 38.6067
R2	0.698	0.541
Ko (h ⁻¹)	0.7276	0.6189
**First-order**		
Equation	y =−0.02172x + 4.1135	Y = -0.02063x + 4.6052
R2	0.8545	0.597
K1 (h ⁻¹)	-0.02172	-0.02063
**Higuchi**		
Equation	y = 9.3167x + 7.0305	Y = 8.5658x + 17.1152
R2	0.896	0.811
KH (μg/h ⁻¹ᐟ²)	9.3167	8.5658
**Korsmeyer–Peppas**		
Equation	y = 0.3914x – 1.8113	Y = 0.1115x – 0.5593
R2	0.9224	0.9845
Km (min ⁻¹)	0.1634	0.5713
n	0.3914	0.1115

Figure S2. Cumulative release behavior and kinetic parameters. The cumulative release (%) of the Mast-Mo peptide from PVA/CS-73 and PVA/CS-91 nanofiber wound dressings, measured at 24, 48, 72, 96, and 120 h, is shown in Figure A. The corresponding kinetic parameters for the release models (zero-order, first-order, Higuchi, and Korsmeyer–Peppas) are shown in Figure B.

Table S1. Characteristic temperatures (T) and enthalpy values (ΔH) of the thermal transitions observed in PVA/CS Mast-Mo nano-fiber mats.

Sample	T peak 1 (°C)	ΔHf_1 (mJ/mg)	T peak 2 (°C)	ΔHf_2 (mJ/mg)
PVA/CS-73	84.4	57.1	190.2	22.7
PVA/CS-73 Mast-Mo7.5	98.1	67.8	189.7	21.3
PVA/CS-73 Mast-Mo15	95.9	144	189.5	24.7
PVA/CS-91	90.4	98.3	192.6	41.5
PVA/CS-91 Mast-Mo7.5	93.1	51.4	189.9	23.1
PVA/CS-91 Mast-Mo15	81.9	134	191.3	32.9
PVA powder	-	-	190.4	40.7

Table S2. Thermogravimetric analysis (TGA) parameters of PVA/CS electrospun membranes loaded with Mast-Mo peptide at different concentrations.

Sample	T_{onset} (°C)	T_{max1} (°C)	T_{max2} (°C)	Char at 700 °C (%)
**PVA/CS-73**	278	328	427	10
**PVA/CS-73 Mast-Mo7.5**	271	325	432	10
**PVA/CS-73 Mast-Mo15**	283	329	431	7
**PVA/CS-91**	248	308	428	8
**PVA/CS-91 Mast-Mo7.5**	262	318	429	9
**PVA/CS-91 Mast-Mo15**	258	318	430	7

T_{onset}: onset degradation temperature; T_{max1}: temperature of maximum degradation rate (Stage 2); T_{max2}: temperature of secondary degradation (Stage 3);). Values marked with ~ are estimated from graphical analysis and should be confirmed with instrument software.

Table S3. Mathematical models used to describe peptide release kinetics from PVA/CS Mast-Mo nanofiber mats.

Model	Equation	Parameters / Description	
Zero-order	$Qt = k0 \cdot t$	Qt = amount of drug released at time t, $k0$ = zero-order release constant.	(EqS1)
First-order	$\ln(Q\infty - Qt) = \ln Q\infty - k1 \cdot t$	$Q\infty$ = initial amount of drug in the nanofiber<br>Qt = amount released at time t, $k1$ = first-order release constant.	(EqS2)
Higuchi	$Qt = kH \cdot t$^1/2	Qt = amount of drug released at time t<br>kH = Higuchi dissolution constant.	(EqS3)
Korsmeyer–Peppas	$Mt / M\infty = kKP \cdot t$^$n$	$Mt/M\infty$ = fraction of drug released at time t<br>kKP = release rate constant<br>n = release exponent indicative of the mechanism	(EqS4)
